# Genome-wide characterization, expression and functional analysis of *CLV3/ESR* gene family in tomato

**DOI:** 10.1186/1471-2164-15-827

**Published:** 2014-09-30

**Authors:** Yu Zhang, Shaohui Yang, Yingjin Song, Jiehua Wang

**Affiliations:** School of Environmental Science and Engineering, Tianjin University, Weijin Rd. 92#, Nankai District, Tianjin, 300072 China

**Keywords:** *Solanum lycopersicum*, *CLE* gene family, Phylogenetic analysis, Tissue expression pattern, Peptide application

## Abstract

**Background:**

By encoding a group of small secretory peptides, the members of the CLAVATA3/EMBRYO-SURROUNDING REGION (CLE) family play important roles in cell-to-cell communication to control the balance between stem cell proliferation and differentiation in plant development. Despite recent identification and characterization of members of this gene family in several plant species, little is known about its functional role in plants with fleshy fruits.

**Results:**

In total, fifteen *CLE* genes (*SlCLE1-15*) were identified from tomato (*Solanum lycopersicum* cv. ‘Heinz-1706’) genome and their multiple characters including phylogeny, gene structures, chromosome locations, conserved motifs and *cis*-elements in the promoter sequences, were analyzed. Real-time PCR analysis showed that 13 out of 15 identified *SlCLE* genes are transcribed and exhibit remarkably unique expression patterns among tissues and organs. In particular, *SlCLE12*, the homologue of *Arabidopsis CLE41/44* gene, appears to be the dominant *CLE* gene in most of tested tissues with its maximum expression found in vascular tissues. Meanwhile, *SlCLE1, 10, 13* exhibit specific but distinct expression in flower bud, root and shoot apex, respectively. More notably, several *SlCLEs* are dramatically regulated in their transcriptional levels during fruit development and ripening, indicating significant role these genes may potentially play in the critical physiological process. Upon the treatment with synthetic peptides corresponding to the 12-aa CLE domains of SlCLE 10, 12 and 13, tomato seedlings exhibit a clear reduction in root length to varying degrees.

**Conclusions:**

This study provides a comprehensive genomic analysis of *CLE* gene family in tomato, a crop species with fleshy fruit. Differential expression patterns of various *SlCLEs* provide important insights into the functional divergence of CLE signaling cascade in *Solanaceae* species, especially their potential involvements in the regulation of fruit development and ripening.

**Electronic supplementary material:**

The online version of this article (doi:10.1186/1471-2164-15-827) contains supplementary material, which is available to authorized users.

## Background

For multicellular organisms, intercellular communication is essential for coordinating their well orchestrated growth and development. In higher plants, besides the conventional phytohormones such as auxin and cytokinin, a group of small secretory peptides, namely the CLAVATA3/EMBRYO-SURROUNDING REGION-RELATED (CLV3/ESR, CLE) peptides, has been recognized as important players that are responsible for mediating cell-to-cell signaling.

CLE proteins usually include a signal peptide at the N-terminus followed by a variable domain and a conserved CLE domain composed of 12–14 amino acids at the C-terminus [1–3]. When exogenously applied, synthetic peptides corresponding to the CLE domains could reduce the size of the shoot apex meristem (SAM) or the root length, or inhibit tracheary element differentiation [1, 2, 4]. With 32 CLE members in *Arabidopsis thaliana* genome, synthetic peptides encoded by at least 19 of them have been shown to be functional [1, 2, 4–6]. Recent work indicated that CLE peptides are involved in many aspects of plant growth and development, including plant-pathogen interaction, cell division, anther-stigma interaction and stem cell maintenance [7]. Representing a founding member of the CLE family, CLAVATA3 (CLV3) has been identified as a key regulator of stem cell homeostasis at the SAM through suppressing the homeodomain transcription factor, WUSCHEL (WUS) [8–11]. In roots, CLE40 peptide functions to maintain the quiescent center (QC) and columella stem cell identity by repressing the WUS-related gene, *WOX5* [12]. In vascular tissues, TRACHEARY ELEMENT DIFFERENTIATION INHIBITORY FACTOR (TDIF) encoded by *CLE41* and *CLE44* promotes the accumulation of undifferentiated procambial cells and inhibits their differentiation into xylem [2]. CLE peptides also take additional biological roles beyond stem cell regulation. For example, CLE8 has recently been identified as a regulator of seed development [13]. Moreover, CLEs have been identified as signaling ligands in plant responses to environmental cues. Two CLE peptides, *Lotus japonicus* CLE ROOT SIGNAL1 and 2 (LjCLE-RS1 and LjCLE-RS2) have been identified as root-derived infection signals that are transmitted from roots to shoots and systemically repress excess nodulation [14]. Although that CLE signaling plays an important role during plant development and in responses to various environmental stimuli, its role in fruit formation and ripening process has yet to be investigated.

Genes encoding CLE peptides and their receptors have been found in a diverse range of plant species [15–17] including monocots (*Oryza sativa* and Zea *mays*), dicots (*Glycine max*, *Populus trichocarpa*, *Medicago truncatula*) and even the moss *Physcomitrella patens* and the alga *Chlamydomonas reinhardtii* [18], thus suggesting that CLE signaling pathway has been conserved during the course of plant evolution [19]. However, the organization and roles of CLEs in crops, especially in fruit-bearing plants has fallen far behind *Arabidopsis*. Therefore, we chose tomato (*S. lycopersicum*), as a model crop with very high economic impact to understand the similarity and diversity of CLE cascade among various plants. Taking advantage of the available online database, we first carried out a genome-wide search for the *CLE* family members in tomato and then investigated their tissue-specific expression patterns. We also investigated the roles of CLE signaling in tomato by synthesizing the 12-aa CLE motif peptides of three SlCLEs specifically expressed in vegetative tissues and observed the effects of their exogenous application on tomato seedlings. Data presented here shed light on the evolutionary conservation of *CLE* gene families across various plant species as well as the various signaling components of CLE pathway in fleshy fruit-bearing crops.

## Results and discussion

### Members and phylogenetic analysis of *CLE*gene family in tomato

We performed a systematic analysis of the tomato CLE small signaling polypeptide family to expand our insights into the developmental processes that are potentially regulated by CLE peptide-mediated signal transduction pathways. Many small proteins including potential ligand peptides, are frequently not detectable by automated annotation programs due to their sizes [16]. To determine the *CLE* family members in tomato, all previously reported CLE proteins from various species were used as query sequences to perform full-length BLASTP searches against tomato proteome database at Phytozome v9.1 [20, 21]. As results, 15 apparent *SlCLE* genes were identified (Additional file 1). Considering the bigger size of *Arabidopsis CLE* gene family (32 members), this low yield could be due to the poor sequence conservation outside the CLE domains and there remained a chance to identify few more CLE family members in tomato genome.

**Figure 1:**
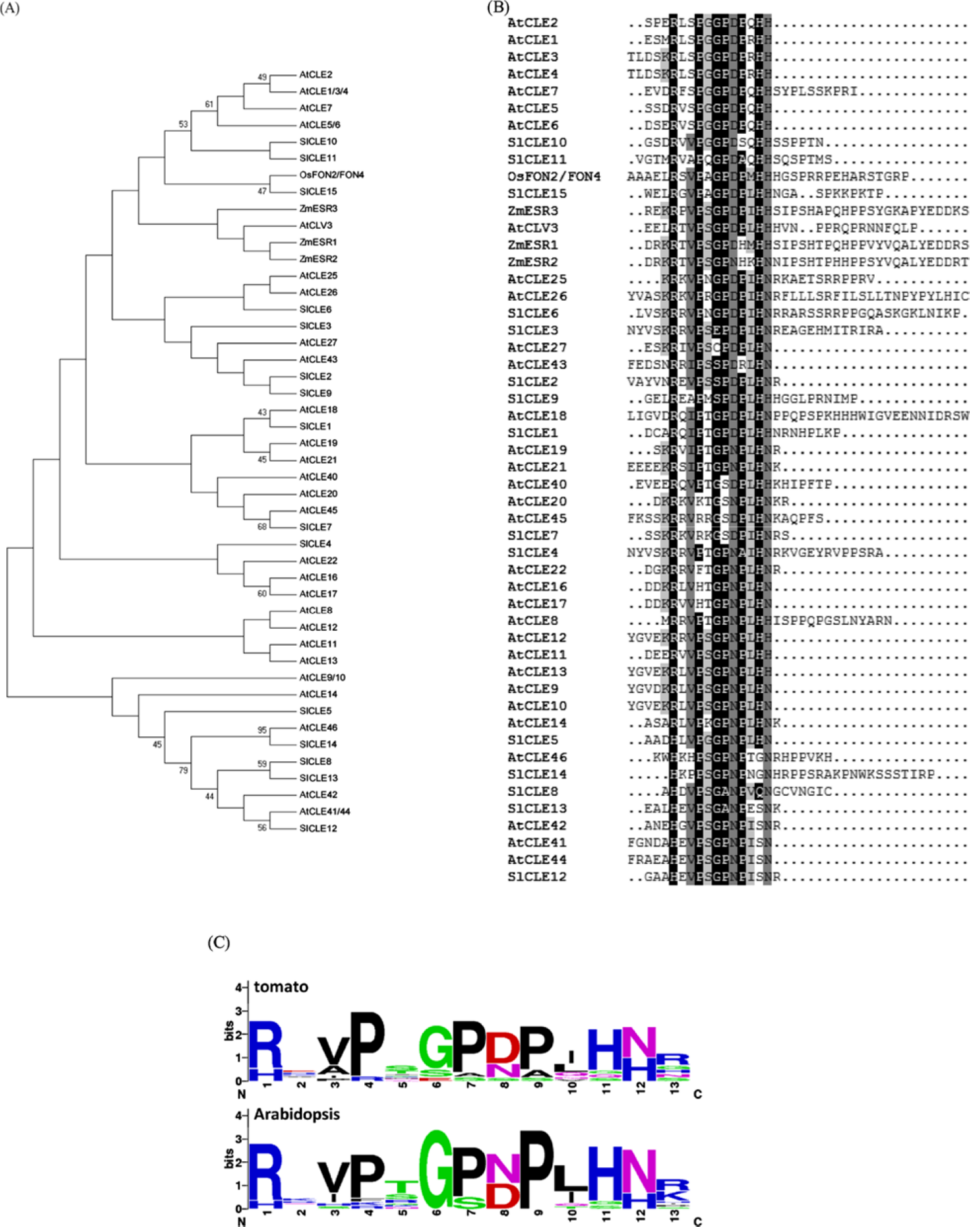
**Phylogenetic tree and sequence analysis of tomato and** ***Arabidopsis CLE*** **gene families. (A)** Maximum likelihood bootstrap tree phylogeny based on the CLE motif sequences of *SlCLE* genes in tomato. The unrooted tree was constructed using MEGA 5.05. Numbers at nodes indicate the percentage bootstrap scores and only bootstrap values higher than 40% from 1,000 replicates are shown. Sl, *S. lycopersicum*; At, *A. thaliana*; Zm, Z. *Maize*. **(B)** Predicted protein sequences of SlCLEs were aligned with AtCLEs with flanking sequences using ClustalX and the output was displayed with Box Shade. **(C)** Sequence logos for the CLE motifs of tomato and *Arabidopsis CLE* gene family members. The height of the bars indicates the number of identical residues per position.

We then examined the phylogenetic relationship of the putative SlCLE family members to *Arabidopsis* CLEs and certain well-known CLE proteins from other species. Because the majority of SlCLE family members have very divergent amino acid sequences outside the CLE motifs, their phylogenetic relationship was not well supported (Figure 1A). However, some branches in the tree were supported by high bootstrap values and thus indicated the most closely related CLE proteins. We are confident of the close relationship of SlCLE15 (Solyc11g071380) to FLORAL ORGAN NUMBER2 and 4 (OsFON2/FON4), which have been reported to regulate floral meristem size in rice [22, 23]. SlCLE10 (Solyc07g053370) and SlCLE11 (Solyc07g062670) are clustered together with AtCLE1-7, which genes form a single subclade among *Arabidopsis* 32 CLE members. The overexpression of *AtCLE1-7* is known to cause a premature termination of SAM and longer roots as well [24, 25]. Interestingly, the functional forms of the above-mentioned three SlCLEs (SlCLE15, SlCLE10 and SlCLE11) are also the SlCLEs sharing the highest sequence similarity with the well-known AtCLV3 and ZmESR1-3. *CLV3* encodes a stem cell-specific protein that plays a key role in stem cell fate determination through mediating the intercellular communication during *Arabidopsis* development [26, 27]. *ZmESRs* are endosperm-specific genes in maize and are supposed to play roles in the nutrition of the developing embryo and in the establishment of a physical barrier between embryo and endosperm [28]. When compared to the 26 predicted active CLE peptides encoded by *Arabidopsis CLE* genes, a perfect match in tomato was only found for SLCLE12 (Solyc09g061410) with the TDIF peptide encoded by AtCLE41/44. TDIF plays a specific role in xylem differentiation [2] and very recently, its perfect conservation in amino acid sequences has been reported across eight conifer species [29] .

Detailed description of all SlCLE polypeptides is provided in Additional file 1 and summarized in Table 1. Briefly, *SlCLE* genes encode polypeptides of 66–110 aa, with a predicted molecular mass range of 7.1–12.6 kDa. Though the vast majority of known CLE sequences have basic pI values [16], the theoretical pI values of SlCLEs range from 4.78 to 11.06 (Table 1). CLE proteins are synthesized as inactive protein precursors and the active 12 or 13-aa peptide ligands are liberated by proteolysis. Unlike the sequence degeneracy among the AtCLEs, all active SlCLE peptides are different in their sequences. When the sequence logo was built for tomato CLE motifs to visualize the conserved residues, the consensus sequence was slightly different from the one generated for AtCLEs (Figure 1B). Four residues including P(4), P(7), P(9) and H(11) are almost invariant over the whole SlCLE family (Additional file 2). Five predicted SlCLEs (SlCLE5, 8, 12, 13 and 14) diverge from the consensus at the otherwise perfectly conserved Arg residue at position 1 with a His residue. A similar occurrence of V/A at position 3, D/N at position 8 and N/H at position 12 are also observed (Figure 1B). Other positions in the CLE domain are highly variable, such as positions 2, 5, 6, 10 and 13 (Figure 1B). In a previous report, 174 predicted CLE motifs from various plant species were classified into 13 distinct groups based on their CLE domain sequences [4]. The signature amino acids of each group in that work could also be identified for some SlCLEs in this study. For instance, the His at position 1 and the Asn at position 11 of SlCLE12 and 13 were both identified as the group V-specific residues [4]. Four residues upstream of the CLE domain have been recently shown to be required for CLE peptide endoproteolytic processing [30]. In the case of SlCLEs, the upstream short regions adjacent to the CLE motif are highly variable and no similarity as previously reported for *Arabidopsis* CLEs [16] has been detected (Additional file 2). At the C terminus of the CLE domain, addition of an arginine has been shown to cause a decrease of peptide activity [2, 31]. In tomato, five out of 15 CLEs include this arginine residue at the C terminus of their respective CLE domains (Additional file 2).

**Table 1:** **A complete list of 15** ***SlCLEs*** **identified in the present study**

Gene name	Gene locus	Unigene ID	Length (bp)	Protein (aa)	MW (kDa)	PI
*SlCLE1*	Solyc01g014100.2.1		676	75	8.65	6.39
*SlCLE2*	Solyc01g098890.1.1		246	81	8.91	4.78
*SlCLE3*	Solyc02g067550.1.1		672	96	10.47	9.61
*SlCLE4*	Solyc02g087470.2.1		516	69	7.62	11.06
*SlCLE5*	Solyc03g025960.1.1	SGN-U566466	201	66	7.13	10.02
*SlCLE6*	Solyc05g006610.2.1	SGN-U599368	2858	110	12.60	10.87
*SlCLE7*	Solyc05g007650.1.1		252	83	9.25	10.14
*SlCLE8*	Solyc05g053630.1.1	SGN-U575421	270	89	9.72	9.57
*SlCLE9*	Solyc06g074060.1.1		403	74	8.40	9.51
*SlCLE10*	Solyc07g053370.1.1		264	87	9.83	6.90
*SlCLE11*	Solyc07g062670.1.1	SGN-U581442	267	88	9.94	6.89
*SlCLE12*	Solyc09g061410.1.1	SGN-U574832	318	105	11.67	8.86
*SlCLE13*	Solyc09g091810.1.1	SGN-U566187	264	87	9.48	8.89
*SlCLE14*	Solyc11g066120.1.1	SGN-U600211	568	95	10.71	10.45
*SlCLE15*	Solyc11g071380.1.1		555	93	10.60	9.63

Genomic DNA sequences are obtained from Phytozome v9.1 (http://www.phytozome.net) (Additional file 1). Amino acid sequences are deduced from the corresponding coding sequences (Additional file 1).

### Gene structures, chromosomal locations, motif analysis and *cis*-elements in promoter regions of *SlCLEs*

**Figure 2:**
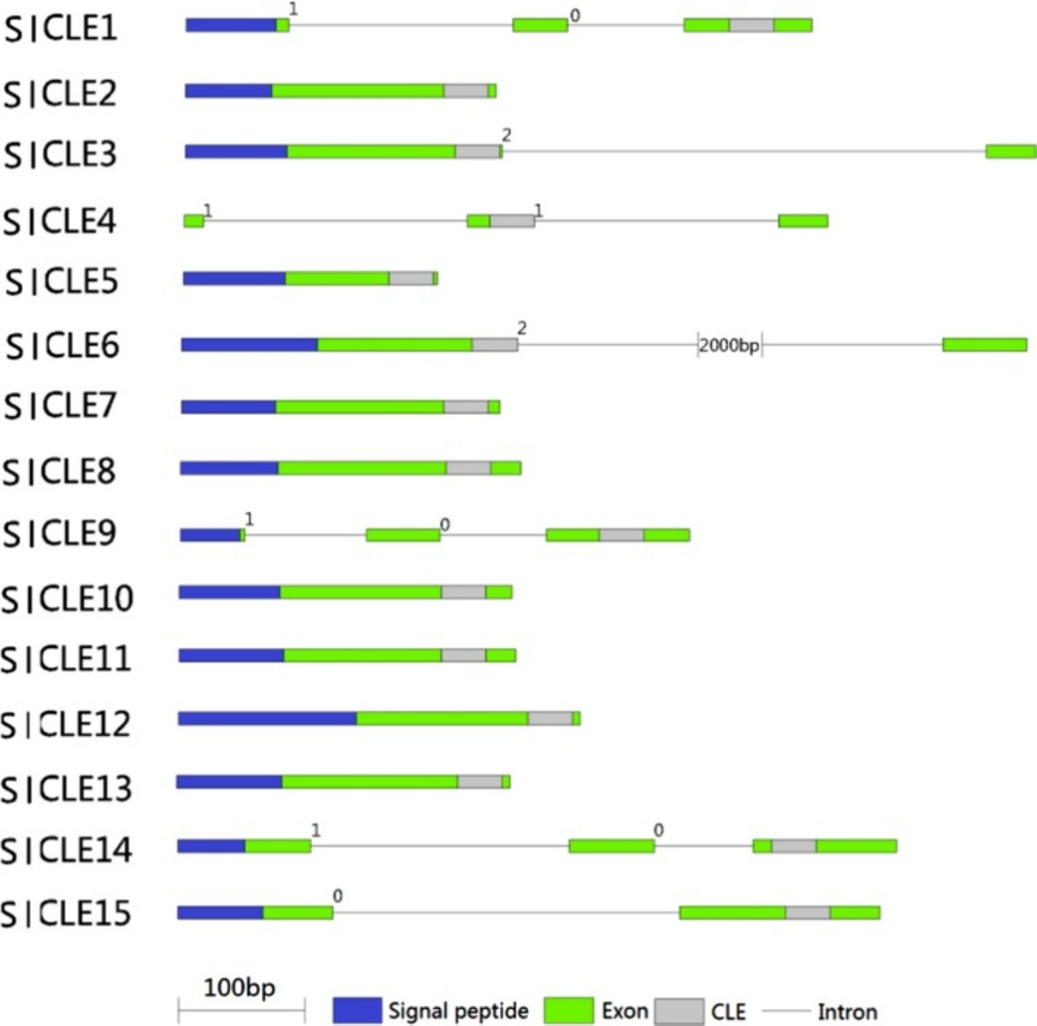
**Schematic diagram of genomic structures of tomato** ***CLE*** **family members.** Exons are shown as green boxes and introns as grey lines. The blue and grey boxes mark the signal peptides and CLE domains, respectively.

Analysis of the predicted SlCLEs suggests that they are all secreted proteins, with signal peptides as predicted by SignalP. The presence of an intron in the sequence upstream of the CLE domain has been reported in *Arabidopsis* for CLE40 and CLV3 [9, 32] and in rice, 9 out of 44 *OsCLE* genes include introns [16]. In tomato, 7 out of 15 SlCLE coding sequences were interrupted by a single or two introns (Figure 2). By contrast, most *Arabidopsis CLE* genes consist of one exon with a single open reading frame [20]. As to the chromosome distribution, 15 *SlCLE* genes are distributed over 8 out of 12 tomato chromosomes with 1–3 genes per chromosome (Additional file 3). Although neighboring *SlCLE* genes could be found in pairs on chromosome 5, 7 and 11, they only share 9.7-26.1% identity within pairs and thus could not be taken as tandem segmental duplication event (Additional file 4). By contrast, whole genome duplication and reshuffling as well as localized gene duplication followed by gene retention have been reported to all contribute to the expansion of *CLE* gene family in *Arabidopsis* during evolution [33], which might explain the fairly large size of *Arabidopsis CLE* gene family.

The motif distribution in tomato and *Arabidopsis* CLE proteins were analyzed using Multiple Expectation Maximization for Motif Elicitation (MEME) tool. Despite that multiple CLE domains have been reported for two *OsCLEs* [17], which implied a possibility of generation of multiple CLE peptides from one polypeptide precursor, no more than one CLE motif was observed within any of the SlCLEs. Besides the CLE motif, another one extremely conserved motif enriched with hydrophobic amino acids is found in the signal peptide regions of SlCLEs (Additional file 5). Except for these two motifs, no other consensus motif was found to be shared by the majority of SlCLEs. However, a close motif comparison between tomato and *Arabidopsis* CLEs revealed that individual SlCLE does share specific motifs with certain AtCLE, such as SolycCLE1 and CLV3 (Additional file 5).

The search for putative *cis* elements within the 2 kb genomic sequences upstream of the 5’-UTR of *SlCLEs* was performed in the PLACE database (http://www.dna.affrc.go.jp/PLACE/signalscan.html). A total of 200 putative *cis* elements were identified and only 29 out of them were able to be found in all promoter regions, suggesting the very complex regulatory behaviors of the *SlCLE* family genes (Additional file 6). Most putative *cis* elements could be classified into four major types. The first type include the elements that are regulated by one of the most important developmental signals, hormones, including abscisic acid (ABA), gibberellins (GA), auxin, jasmonic acid (JA) and ethylene (ETH). For example, the GADOWNAT sequence similar to ABRE was found to be present in 24 GA down-regulated genes involved in *Arabidopsis* seed germination [34]. Diverse *cis*-elements responsive to hormones identified in the promoters of *SlCLE* genes indicate that CLE peptide signaling transduction pathway interacts dynamically with other phytohormone signaling pathways. C*is*-elements of the second type identified are either related to organgenesis or required for tissue-specific gene expression patterns, which make them very attractive considering the important roles played by CLEs in plant meristem maintenance and organogenesis. For example, the core AACA motifs found in rice glutelin genes are involved in controlling their endosperm-specific expression [35] and the TGA1a motif is likely to contribute to the root tip meristem-specific expression of GST isoenzymes [36]. C*is*-elements of the third type are light-related ones, including the GT1 CONSENSUS binding site present in many light-regulated genes [37]. C*is*-elements of the fourth type are generally related to environmental stimulus including both abiotic and biotic stresses and this type of elements outnumbered other element categories except the light regulation-related type, which indicates the involvement of SlCLEs in stress responses. In addition, two very interesting elements, WUSATAg and XYLAT could be found in 5 (*SlCLE11, 2, 7, 13* and *12*) and 2 (*SLCLE3* and *6*) genes, respectively, with 1 or 2 copy numbers. The WUSATAg has been identified as a target sequence of WUS in the intron of *AGAMOUS* gene in *Arabidopsis* [38]. In both SAM and RAM, the interplay between *CLE* genes and different *WUS* gene lays the foundation of meristem cell population regulation. The XYLAT element is a *cis*-element characterized among the promoters of the “core xylem gene set”, reminding us of the TDIF, which is highly relevant to the xylem cell differentiation [39].

### *SlCLE*expression patterns indicate their involvement in many developmental processes including fruit ripening

**Figure 3:**
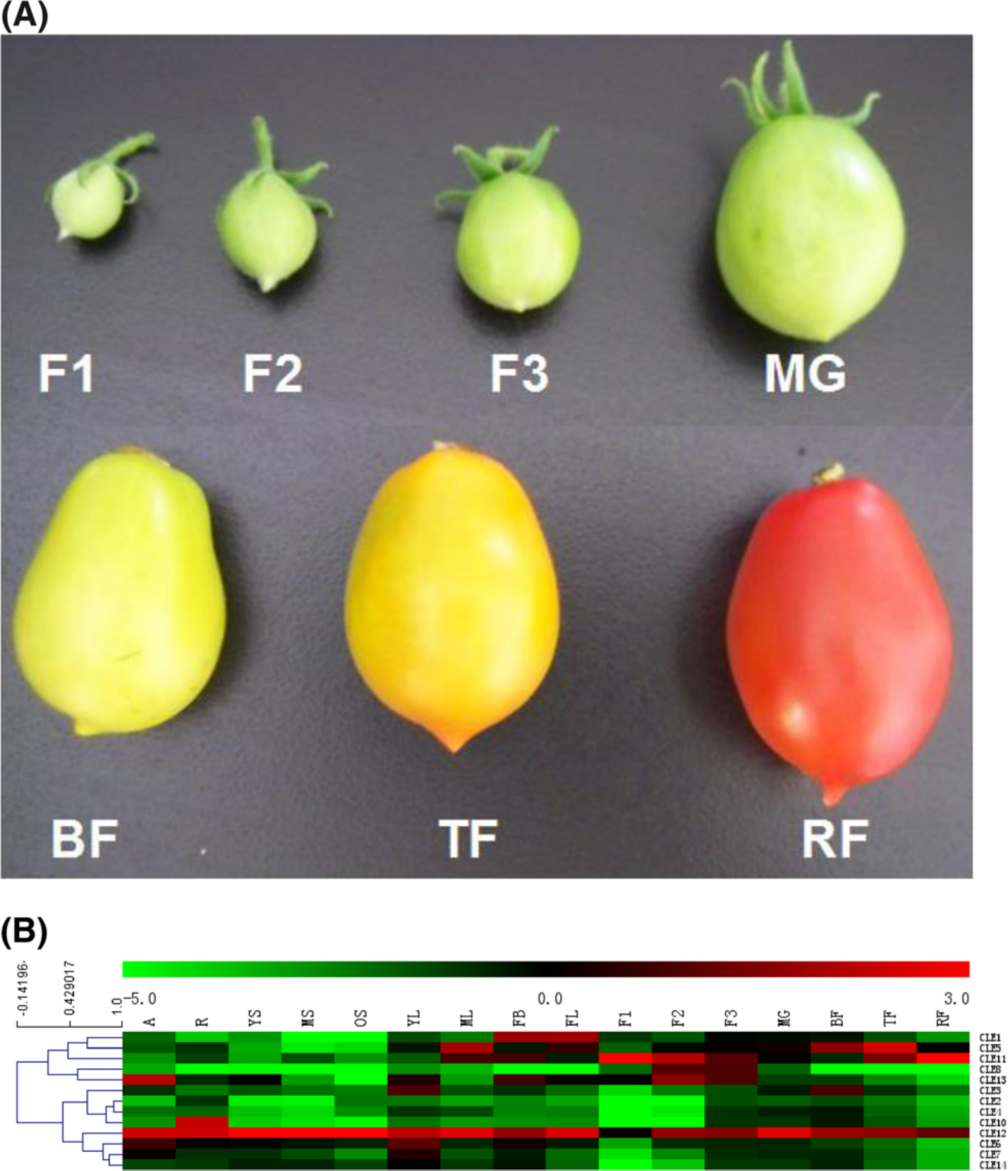
**Expression patterns of** ***SlCLE*** **genes in selected tissues and fruits at different developmental stages. (A)** Photos of tomato fruits at seven developmental stages assessed. **(B)** Transcription levels of *SlCLEs* investigated by RT-qPCR shown as heat-map for better illustration. Relative gene expression levels were normalized to have mean zero and variance one with actin transcript values before producing the heat maps. The RT-qPCR analyses were repeated at least three times in three independent experiments. Color scale at the top of each dendrogram represents log2 values. Total RNA isolated from apex (A), root (R), young, medium and old stems (YS, MS, and OS), young and mature leaf (YL and ML), flower (FL), flower buds (FB), and 1-cm, 2-cm and 3-cm sized green fruits (F1, F2 and F3), mature green fruit (MG), fruit at breaker stage (BF), fruit turning red (TF) and mature fruit in red(RF).

**Figure 4:**
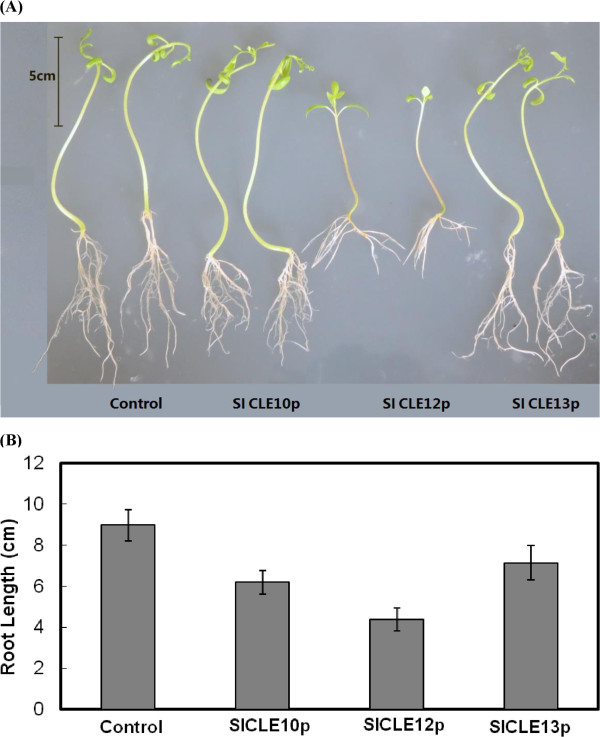
**Phenotypes of 10-d tomato seedlings upon the treatment of SlCLE motif peptides. (A)** Effect of 100 *μ*M concentration of SlCLE10p, 12p and 13p on 10-day old tomato seedling development. **(B)** Effects of different SlCLE motif peptides on the root length of tomato seedlings. The lengths of the main roots were measured after 10 d of growth on peptide-containing media (n = 25 for each treatment). Data and error bars represent mean ± SD.

In *Arabidopsis*, the specificity of CLE function has been proposed to be mainly achieved by differential expression patterns of *CLE* genes. In order to gain insights into the potential biological roles of *SlCLEs*, we analyzed their temporal and spatial expression profiles in various tissues and fruits at different developmental stages using qRT-PCR (Additional file 7 and Figure 3). In *Arabidopsis*, all but one of the 32 *AtCLE* genes are transcribed [33]. In tomato, thirteen out of fifteen *SlCLEs* were able to be cloned from a pooled cDNA derived from an array of tomato tissues. All tissues examined express multiple *SlCLE* genes and the overlap among different *CLE* gene expression patterns was conspicuous (Additional file 7 and Figure 3). In brief, the expression level of all *SlCLEs* is low in small green fruit and increases after the breaker stage (Additional file 6 and Figure 4). *SlCLE2*, *3*, *4*, *6*, *7*, and *14* exhibit a relatively uniform expression among the examined tissues; however, the remaining *SlCLEs* exhibit a quite differential expression pattern among tissues and organs (Additional file 7 and Figure 3). Notably, *SlCLE12* appears to be the most abundantly expressed *SlCLE* in almost all tested tissues except for the green fruit of 1 cm size (Additional file 7 and Figure 3). In *Arabidopsis*, an exactly same CLE peptide (TDIF) as SlCLE12p and a very similar one are produced by *CLE41/44* and *CLE42*, respectively [2, 40]. Using the GUS (β-glucuronidase) reporter gene assay, *CLE41*/*44* are found to be expressed preferentially in vascular bundles, while *CLE42* is expressed strongly in SAM and axillary meristems. Therefore, CLE41/44 and CLE42 have been suggested to play roles in regulating wood development [41–43] and the meristematic activity [44], respectively. Considering its vascular-specific expression, SlCLE12 could be most likely denoted as the orthologue of CLE41/44 in tomato; however, its broad and significant expression in a range of tissues and organs imply an even higher functional diversity or a basic function of TDIF in regulating biological processes. As an evidence, a novel function in enhancing axillary bud formation has been recently disclosed for *Arabidopsis* TDIF peptide [44].

*SlCLE1*, *SlCLE10*, *SlCLE12* and *SlCLE13* are specifically expressed at a high transcript level in flower bud, root, stem and shoot apex, respectively, suggesting that they might play particular roles during the development of these organs (Additional file 7 and Figure 3). The *Arabidopsis* counterpart of *SlCLE1* is *AtCLE18* (At1g66145) and the synthetic 12-aa peptide derived from the CLE18 motif has been shown to suppress root growth and cause a short-root phenotype when applied exogenously [2]. *SlCLE13* is highly and specifically expressed in tomato shoot apex, where *Arabidopsis* CLV3 plays a critical role in the maintenance of stem cell reservation. However, since they only share low sequence similarity, they might not be the functional equivalence. *SlCLE5*, *8* and *11* reveal their expression peaks in fruit indicating their potential involvements in fruit development and ripening process. In particular, the transcript level of *SlCLE8* sharply increases in green fruit at 1 cm stage and continuously increases during early fruit development and dramatically declines when green fruit reaches maturation (Additional file 7 and Figure 3). It is noteworthy that despite of its attracting green-fruit expression specificity, the transcript level of *SlCLE8* is lower than that of *SlCLE11*, *12* and *13*, which are the dominant *SlCLEs* in green fruit (Additional file 7 and Figure 3). With its maximum expression found in stem tissue, *SlCLE12* is still the most highly expressed *SlCLE* gene in green fruit until its transcript level is surpassed by *SlCLE5* and *SlCLE11* in orange and red ripe fruits, respectively (Additional file 7 and Figure 3). *SlCLE5* reaches its maximum transcription level at the turning stage and keeps a high expression level together with *SlCLE11* in red ripe fruit, indicating their roles in later process of fruit and/or embryo/seed development. Based on these results, we propose a hypothesis that *SlCLEs* play important and sequential roles in the fruit ripening process. It has been reported that receptors of CLE peptide including CLV1, CLV2, and CRN function together in meristems and gynoecia to regulate *Arabidopsis* fruit organ number and the authors also suggested that CLE ligand(s) other than CLV3 is acting in the CLV pathway in gynoecia [45]. The results shown here further support the importance of the CLE-mediated signaling network in regulating organogenesis throughout plant life cycle and may provide information about key players in the regulation of fruit quality in tomato and other crop plants.

In the original tomato genome sequencing report, the relative expression of all tomato genes was determined by replicated strand-specific Illumina RNA-Seq using tissues including root, leaf, flower (2 stages) and fruit (6 stages) [46]. Based on their data, we performed an *in silico* gene expression analysis of tomato *CLE* gene family members. Similar expression patterns as shown by the qPCR results were identified for some *SlCLE* genes (Additional file 8). For example, *CLE5* and *CLE11* are highly expressed in orange and red fruit at maturation, while *SlCLE7* and *13* are preferentially expressed with abundance in flower buds (Additional file 8). However, there are also some discrepancies between the RNA-sequencing work and the results we obtained by qPCR method. For example, we did not confirm the reported high expression level of *SlCLE2*, *4*, *6*, *14* in root (Additional file 8); instead, *SlCLE10* appear to be the most specifically and highly expressed *CLE* gene in root (Additional file 7 and Figure 3).

### Effects of synthetic SlCLEs application on tomato seedlings

In literature, the active structure of AtCLV3 has been determined as a 12-aa hydroxylated peptide through matrix-assisted laser desorption/ionization–time-of-flight mass spectrometry (MALDI-TOF MS) [1] and the TDIF-peptide isolated from *Zinnia elegans* mesophyll cell culture is also a dodecapeptide with two hydroxyproline (HyP) residues [2]. Thus, in this work we synthesized the 12–amino acid peptides representing the CLE domains of SlCLE10, 12, 13 with hydroxyzed pralines and used them for *in vitro* application assays since these three *SlCLEs* are most specifically expressed genes in vegetative tissues (root, stem and apex, respectively). Tomato seeds were germinated on 1/2 MS media plates containing individual peptide at 10, 50 or 100 *μ*m and 10 days after germination, a dose-dependent inhibitory effect on root growth was observed. Treatments with SlCLE10, 12 and 13 CLE motif peptides dramatically inhibited the root growth by 31%, 51% and 20%, respectively, when applied at the 100 *μ*M concentrations (Figure 4). These results basically resembled the short root phenotype generated by *in vitro* application of AtCLE peptides on *Arabidopsis* seedlings and gave us the first indication of the functional role played by the motif peptides encoded by SlCLEs. In *Arabidopsis*, application of 19 different synthetic AtCLE peptides has been reported to arrest root meristem growth [2, 24, 47], whereas the application of CLE41/44 (TDIF), CLE42, or CLE43 peptides only caused suppressed xylem differentiation [2, 47]. In a previous work, after growing on CLV3p, CLE19p, and CLE40p-containing media for 7 days, a similar inhibition was found for the lengths of the *Arabidopsis* main roots [6]. In contrast, three synthetic SlCLE peptides in this work produced varied short root phenotypes (Figure 4). Unexpectedly, the root-specific SlCLE10p inhibited the root length to a slighter degree than the TDIF encoded by *SlCLE1*2. In phylogenetic tree, SlCLE10p is positioned in a single clade together with AtCLE1-7 peptides by sharing 10 identical amino acids over the 12-aa CLE motif sequence. Overexpression of AtCLE1-7 in *Arabidopsis* result in the developmental timing delay and *wus*-like phenotypes (premature SAM termination, misshapen leaves, flower abnormalities and long root accompanied with anthocyanin accumulation) [24], but no phenotypes has been reported for roots treated with synthetic peptides corresponding to the CLE2 and CLE4-7 CLE motifs [2]. In contrast, we did not observe any of the above-mentioned *Arabidopsis* phenotypes in the current work and instead, an inhibition on root length was detected upon the application of SlCLE10p, which might indicate the functional variance of SlCLE10p and AtCLE1-7p in two different plant species.

SlCLE12 produces an active CLE motif exactly as same as TDIF, a dodecapeptide corresponding to the CLE motif of *Arabidopsis* CLE41/44. In *vitro*, TDIF could independently suppress the differentiation of procambial cells to treacheary elements and promote cell division, but is unable to terminate the RAM development and cause root development retardation [42]. Overexpression of TDIF has been reported to cause dwarf growth and shrub-like phenotype due to a lack of apical dominance without affecting the growth from the SAM or root elongation [24]. Different from these data, in this work SLCLE12p dramatically reduced the growth rate of primary root when applied to tomato seedlings accompanied with an accumulation of anthocynin in the hypocotyl part (Figure 4). Seven *AtCL*E genes not including *CLE42* or *CLE44* have been reported to cause apparent anthocyanin overproduction when overexpressed in *Arabidopsis* plants and this phenotype promoted the authors to suggest possible roles for CLE family members in the regulation of plant stress responses [24]. Similar to SlCLE 10p and 12p, the application of SlCLE13p on tomato seedlings also suppressed the root length elongation but to a slightest degree (Figure 4). Despite of its apex-predominant expression, no misfunctioning of the SAM and any arrest of organogenesis from the shoot tip was observed upon the application of SlCLE13p. Since SlCLE13p is very similar to SlCLE12p in the amino acid composition except that Pro7 and Ile10 in SlCLE12 are substituted by Ala7 and Glu10 in SlCLE13, respectively, it is intriguing to speculate why they inhibit the root growth differentially. In a previous work, when Pro4 and Pro7 of CLV3 was substituted with sarcosine, the simplest imino acid (N-methylglycine), its inhibition activity on *Arabidopsis* roots was significantly decreased accompanied with a weaker receptor binding activity [48]. In addition, Pro7 is also the posttranslational arabinosylation location of several CLE peptides including CLV3 and this sugar modification strongly enhances its binding to corresponding receptor CLV1 and its biological effect [49]. Therefore, tomato CLE peptides probably also act in a sequence-specific manner as previously reported [6] and the substitution of Pro at position 7 to Ala in SlCLE13p might lead to its decreased activity compared to SlCLE12p.

It is noteworthy that the reduction in root length observed for tomato seedlings treated by SlCLE10p, 12p, or 13p could only be taken as the first sign of their potential physiological roles and by no means limit their role in additional processes or to rule out other SlCLEs as key regulators of growth and development in various parts of plant. In order to uncover the potentially diverse functions and activities of SlCLE peptides, functional analysis by ectopic expression or gene silencing techniques is undoubtedly needed to provide further insights into the molecular properties of CLE peptides and their modes of action in the tomato life cycle, in particular in the events of fruit formation and maturation.

## Conclusions

Characterization of *CLE* genes in a fleshy fruit-bearing crop species would facilitate a better understanding of the functions of CLE signaling cascade. Using tomato as a model, a comprehensive overview of *SlCLE* gene family is presented, including the gene structures, phylogeny, chromosome locations, conserved motifs and *cis*-elements in promoter sequences. qRT-PCR analysis showed that 13 of the 15 *SlCLE* genes are transcribed and multiple *SlCLE* genes are actively expressed in a overlapping manner within a given tissue. Several *SlCLE* genes are tissue-specific expressed and developmentally regulated during fruit organogenesis and maturation. *SlCLE12*, the TDIF homologue in tomato, exhibits an exceptionally high expression level in almost all investigated tissues and organs including fruits at various developmental stages. Synthetic peptides corresponding to the CLE motif of SlCLE10, 12 and 13 could significantly and variably inhibit the elongation of tomato primary root and SlCLE12p also leads to an arrest of shoot growth and anthocynin accumulation in hypocotyl, suggesting that such CLE peptides are likely to be the functional CLE gene products. Data shown in this study would provide a very useful reference for future functional analysis of member of *CLE* genes in *Solanum* crops, one of the largest angiosperm genera.

## Methods

### Gene identification and sequence analysis

To search the tomato *CLE* genes, the amino acid sequences of *Arabidopsis*, rice, poplar and other known CLEs were used to perform BLASTP searches against the Phytozome v9.1 (http://www.phytozome.net/) and the tomato database on the SOL Genomics Network (http://solgenomics.net). The retrieved tomato sequences were used as queries to repeat the step in an iterative manner. The positive hits were characterized with a conserved 12-aa CLE domain at the C-terminus and a hydrophobic signal peptide at the N-terminus. Full-length amino acid sequences (60–120 amino acids) or sequences corresponding to the CLE domains were aligned using ClustalX2.1. Phylogenetic analysis was performed using MEGA5.05 program by the maximum likelihood (ML) method with 1000 bootstrap replicates. MEME utility (http://meme.ebi.edu.au/meme/cgi-bin/meme.cgi) was used to display motifs of SlCLE proteins with default parameters except that the motif width was set from 10 to 300 amino acids and the maximum number of motifs to find was five [50]. To determine the locations of *SlCLE* genes on tomato chromosomes, each sequence was further used as query sequence for the BLASTN search against SGN tomato whole genome scaffolds data (2.30) (http://www.sgn.cornell.edu/tools/blast/). To investigate *cis*-elements in promoter sequences of tomato *CLE* genes, 2000 bp genomic sequences upstream of the initiation codons were analyzed for *cis* regulatory elements at the PLACE website (http://www.dna.affrc. go.jp/PLACE/) [51, 52].

### Plant growth and organ-specific expression analysis

Tomato seeds were sown and grown in soil for 5 weeks with a day length of 16 h at a constant temperature of 25°C. The different organs were cut from the plants and total RNA was isolated from fresh tissue using the EasyPure^TM^ Plant RNA Kit (TransGen, China) according to the manufacturer’s instructions and genomic DNA was removed with DNase I treatment. The concentration and quality of the RNA samples were examined using Nanodrop 2000 spectrophotometer (Thermo Fisher Scientific, Finland). One *μ*g of total RNA was used to synthesize cDNA with the oligo-(dT)_18_ primer using the EasyScript® First-Strand cDNA Synthesis SuperMix (TransGen, China). Quantitative Real-Time PCR (qRT-PCR) analysis of cDNA was performed on a PikoReal 96 Real-time Thermal Cycler and PikoReal Software (V2.2) (Thermo Fisher Scientific, Finland) using SYBR® Premix Ex Taq^TM^ (TaKaRa, Japan) and specific primers shown in Additional file 9. The following thermal cycle conditions were used: 95°C for 2 min, followed by 45 cycles of 95°C for 20 s and 58°C for 20 s, 72°C for 30 s. All reactions were performed in triplicate from three independent pooled samples (4 plants per sample). Relative quantification of specific mRNA levels was analyzed using the cycle threshold (Ct) 2^-ΔΔ^Ct method. Relative expression levels are normalized using the housekeeping gene actin and shown in folds of root expression value. Student’s *t* test (*P* < 0.05) was used to determine the significant difference of relative expression of individual genes among different samples. A gene in a given tissue was defined as preferentially expressed only if the expression value of the gene in this tissue was more than 2-fold and had a *P* value less than 0.05 compared to other tissues. Based on the data, the heat map was generated using the MeV4.9.0 software (http://www.tm4.org/) following the instruction.

### Synthetic SlCLE motif peptide application on tomato seedlings

For peptide treatments, the sterilized seeds were placed on half strength Murashige and Skoog media (Duchefa, Netherlands) containing 10, 50 or 100 *μ*M SlCLE peptides, 1% (w/v) sucrose, with 1.5% (w/v) Daishin agar (Brunschwig Chemie, Netherlands). Plates were first incubated at 4°C in the dark for 2 d and then transferred to a room with a temperature of 22°C, 16 h light per day. Three peptides including SlCLE10p (RVVHypGGHypDSQHH), SlCLE12p (HEVHypSGHypNPISN), SlCLE13p (HEVHypSGANPESN), were ordered from Scilight Biotechnology (Beijing, China) with a purity of >90% and dissolved in a filter-sterilized sodium phosphate buffer (50 mM, pH 6).

### Availability of supporting data

The data sets supporting the results of this article are included within the article and its additional files.

## Electronic supplementary material

Additional file 1: **Nucleic acid sequences and amino acid sequences of all 15 tomato** ***CLE*** **gene family members.** (XLS 40 KB)

Additional file 2: **Multiple alignments of the full-length amino acid sequences of** ***SlCLE*** **gene family members using DNAMAN alignment program.** Black and light gray shading indicates identical and conversed amino acid residues, respectively. (TIFF 140 KB)

Additional file 3: **Locations of** ***SlCLE*** **genes on the tomato chromosomes.** Physical distances are in megabases (Mb) and gene transcription orientations are marked by solid arrows. (DOCX 34 KB)

Additional file 4: **Homology, similarity and identity of** ***SlCLE*** **family members over their full-length sequences.** (DOC 110 KB)

Additional file 5: **Schematic illustrations of the types and distributions of motifs in tomato and** ***Arabidopsis*** **CLE family.** The motif characterization was based on full-length proteins by MEME web server. (XLS 1 MB)

Additional file 6: The variety and numbers of *cis*-acting elements in the 2 kb regions upstream of *SlCLE* genes searched in the PLACE database (http://www.dna.affrc.go.jp/PLACE/signalscan.html). (XLS 132 KB)

Additional file 7: **Tissue-specific transcription levels of 13** ***SlCLEs*** **using qRT-PCR method.** The expression levels of individual *SlCLE* gene among various tissues/organs as well as multiple *SlCLE* genes within one individual tissue/organ were presented. Relative gene expression levels are normalized with actin transcript values. The RT-qPCR analyses were repeated at least three times in three independent experiments. Total RNA isolated from apex (A), root (R), young, medium and old stems (YS, MS, and OS), young and mature leaf (YL and ML), flower (FL), flower buds (FB), and 1-cm, 2-cm and 3-cm sized green fruits (F1, F2 and F3), mature green fruit (MG), fruit at breaker stage (BF), fruit turning red (TF) and mature fruit in red (RF). (DOC 86 KB)

Additional file 8: **Normalized expression of tomato** ***SlCLE*** **genes based on RNA-seq data downloaded from previously published data** [46]. Based on the data, the heat map was generated using the MeV4.9.0 software (http://www.tm4.org/) and gene-wise normalized and hierarchical clustered based on Pearson correlation. Color scale at the top of each dendrogram represents fold change values. Bud: unopened flower buds; flower: fully opened flowers; MG fruit: mature green fruit; B_fruit: fruits at breaker; B + 10_fruit: fruits at breaker + 10 days stage. (ZIP 15 KB)

Additional file 9: **qRT-PCR primers used in the current study.** (XLS 27 KB)
