# Supplementary figures and images for: Genome-wide characterization, expression and functional analysis of CLV3/ESR gene family in tomato
Source: BMC Genomics. 2014 Sep 30;15(1):827. doi: 10.1186/1471-2164-15-827 (PMC4195864; doi:10.1186/1471-2164-15-827)

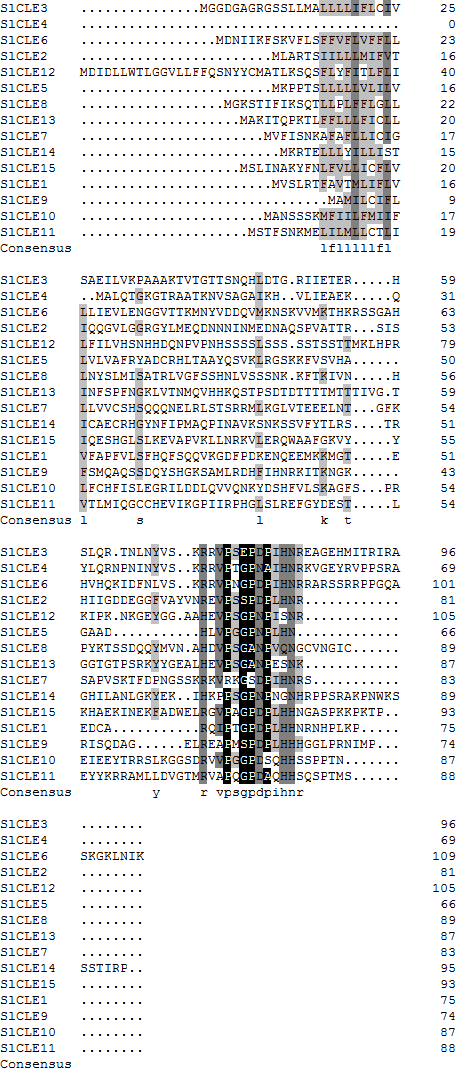

Supplement: Supplementary file 2 — Additional file 2: Multiple alignments of the full-length amino acid sequences of SlCLE gene family members using DNAMAN alignment program. Black and light gray shading indicates identical and conversed amino acid residues, respectively. (TIFF 140 KB) [file 12864_2014_6524_MOESM2_ESM.tiff]

**Additional file 3.**


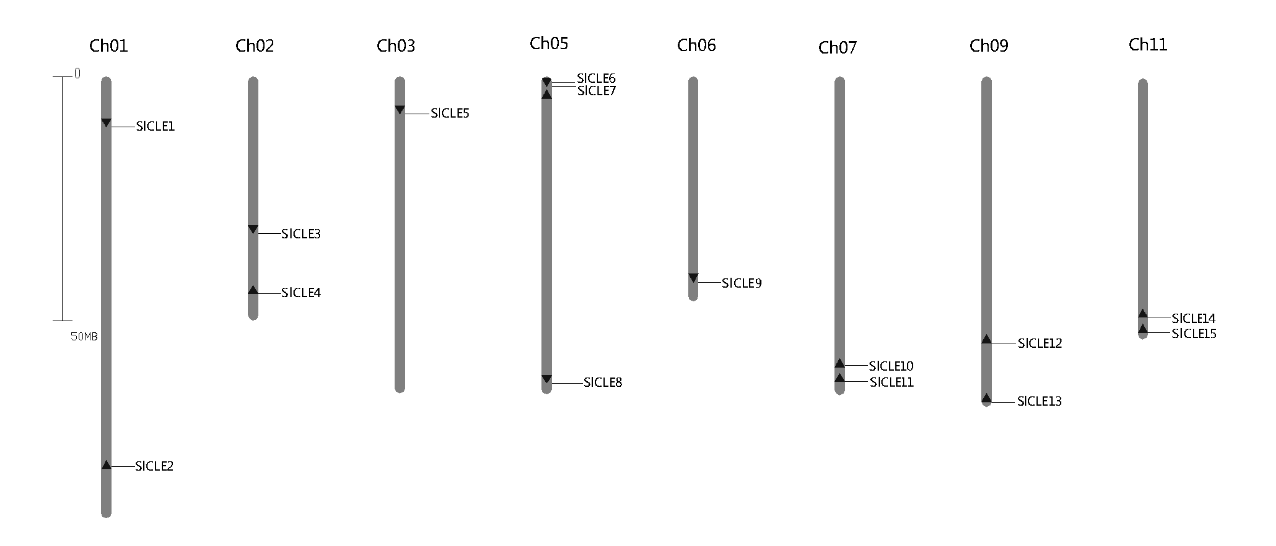

Supplement: Supplementary file 3 — Additional file 3: Locations of SlCLE genes on the tomato chromosomes. Physical distances are in megabases (Mb) and gene transcription orientations are marked by solid arrows. (DOCX 34 KB) [file 12864_2014_6524_MOESM3_ESM.docx]

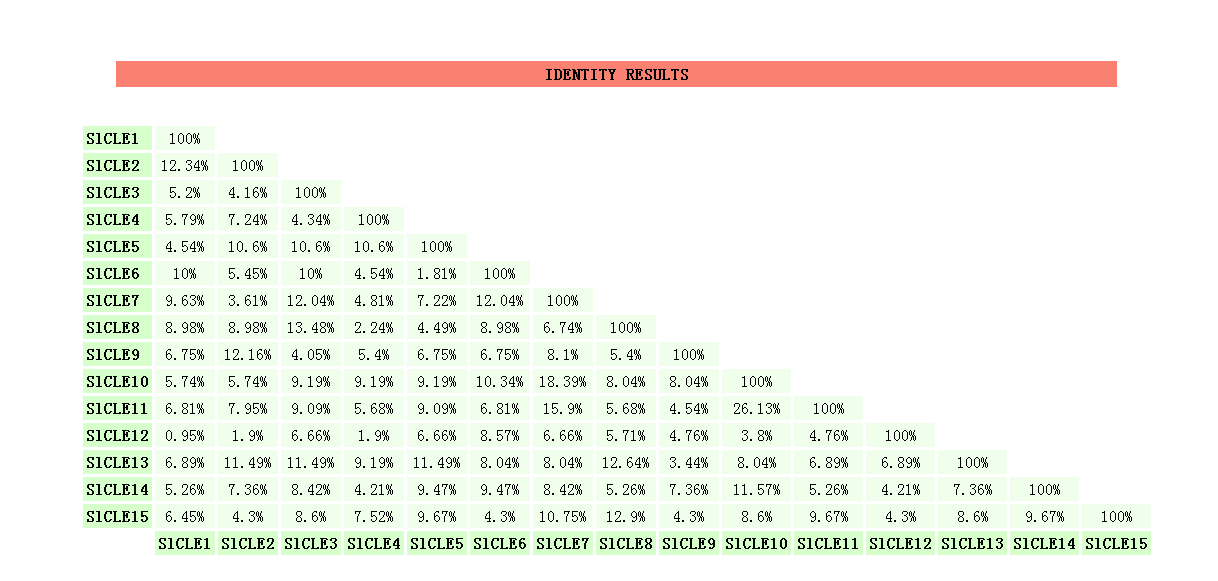

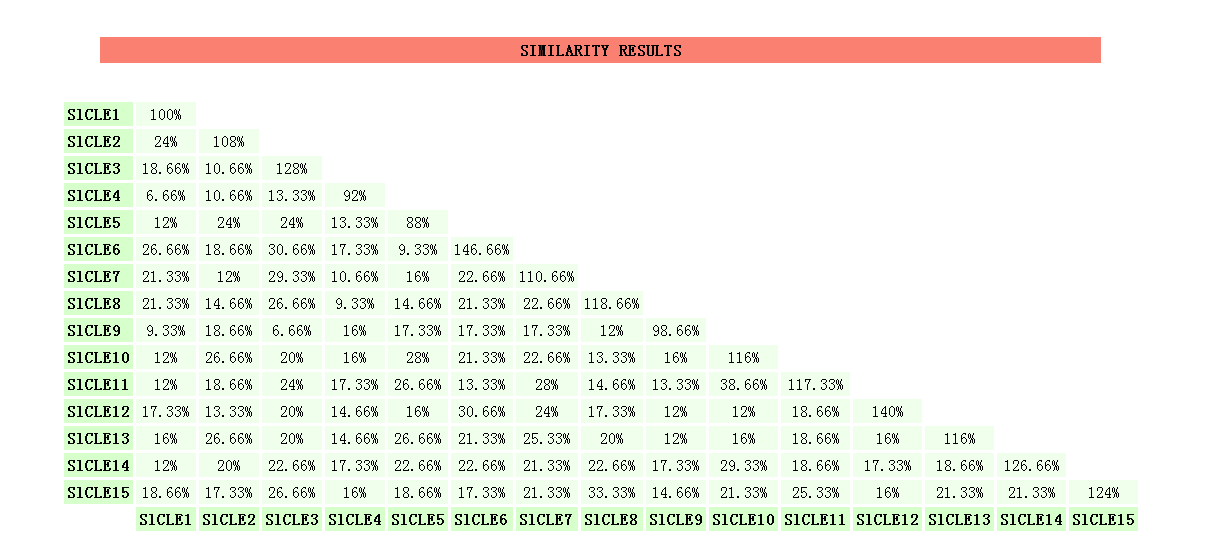

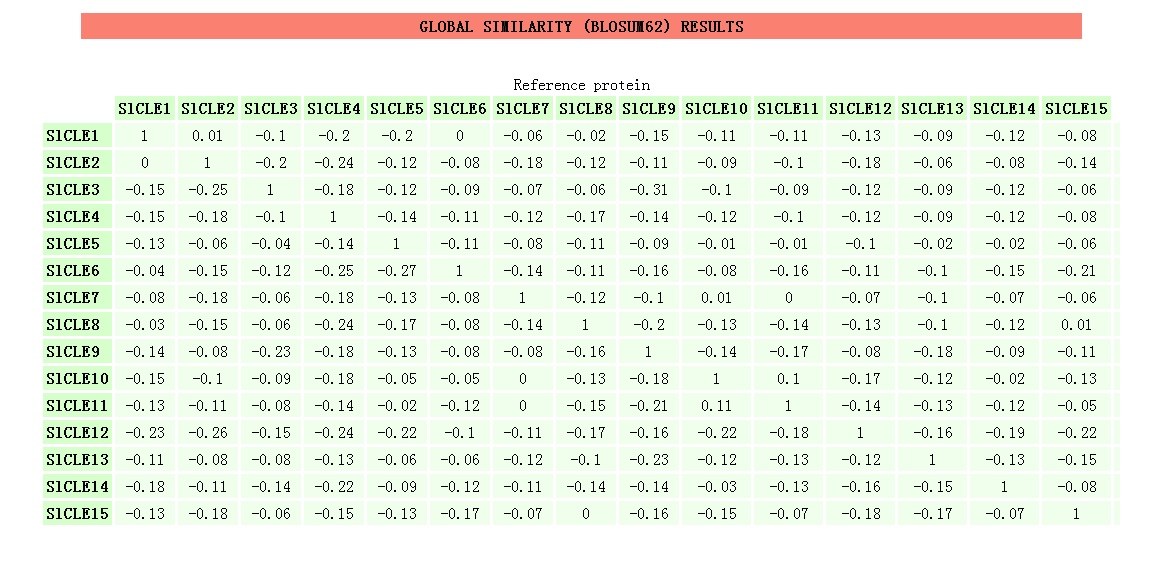

Supplement: Supplementary file 4 — Additional file 4: Homology, similarity and identity of SlCLE family members over their full-length sequences. (DOC 110 KB) [file 12864_2014_6524_MOESM4_ESM.doc]

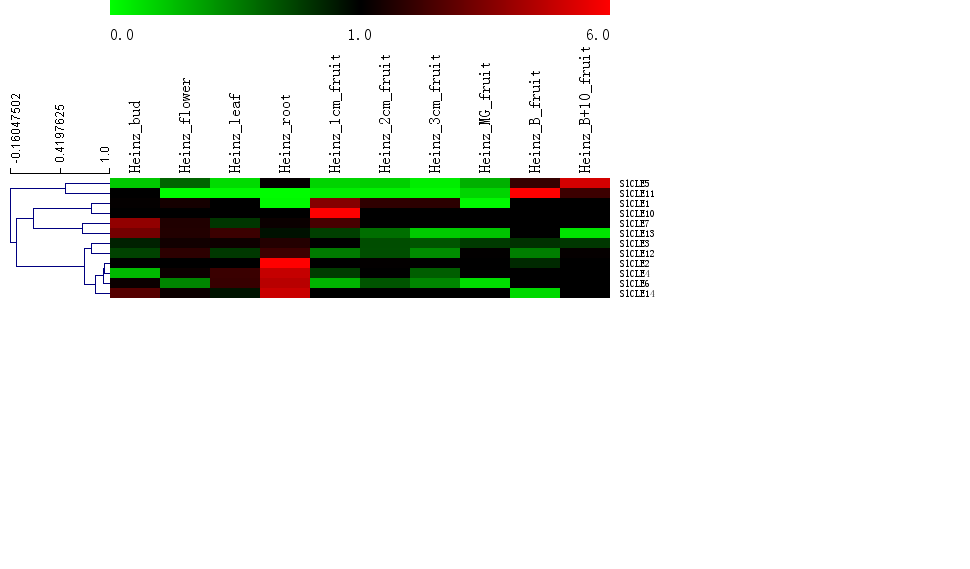

Supplement: Supplementary file 8 — Additional file 8: Normalized expression of tomato SlCLE genes based on RNA-seq data downloaded from previously published data [46]. Based on the data, the heat map was generated using the MeV4.9.0 software (http://www.tm4.org/) and gene-wise normalized and hierarchical clustered based on Pearson correlation. Color scale at the top of each dendrogram represents fold change values. Bud: unopened flower buds; flower: fully opened flowers; MG fruit: mature green fruit; B_fruit: fruits at breaker; B + 10_fruit: fruits at breaker + 10 days stage. (ZIP 15 KB) [file 12864_2014_6524_MOESM8_ESM.zip › 1597736214129100_MOESM8_ESM.bmp]
