# Supplementary material for: Genome-wide characterization, expression and functional analysis of CLV3/ESR gene family in tomato
Source: BMC Genomics. 2014 Sep 30;15(1):827. doi: 10.1186/1471-2164-15-827 (PMC4195864; doi:10.1186/1471-2164-15-827)
Supplement: Supplementary file 3 [file 12864_2014_6524_MOESM7_ESM.doc]

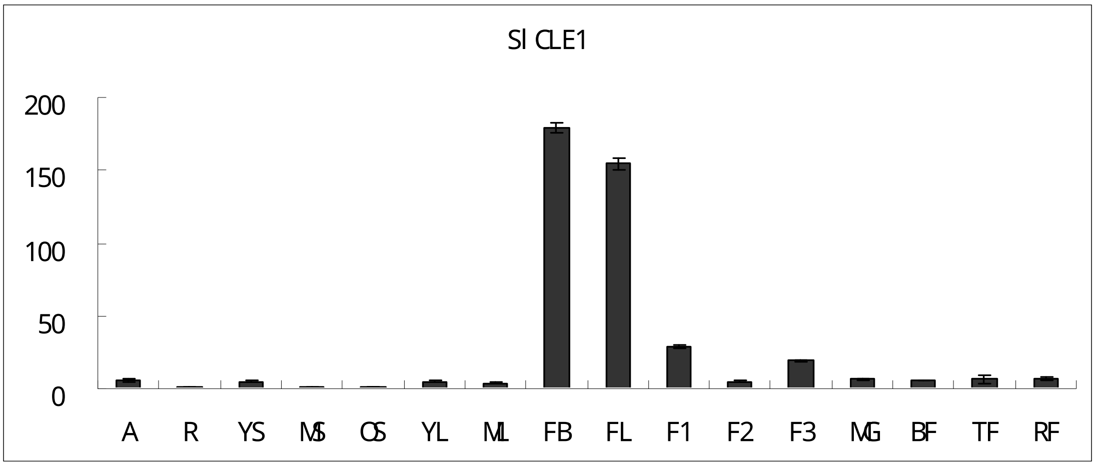

Sl CLE1
200
150
100
50
0
A R YS MS OS YL ML FB FL F1 F2 F3 MG BF TF RF

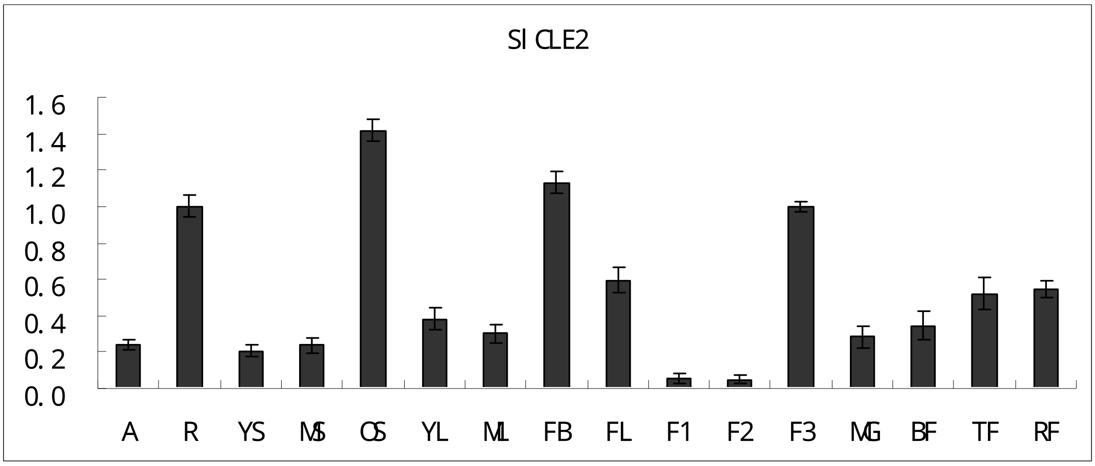

Sl CLE2
1.6
1.4
1.2
1.0
0.8
0.6
0.4
0.2
0.0
A R YS MS OS YL ML FB FL F1 F2 F3 MG BF TF RF

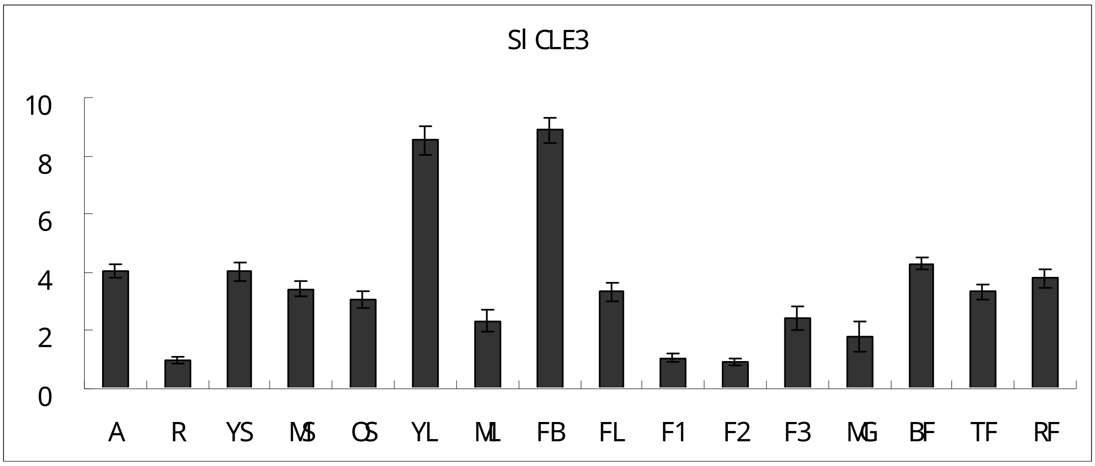

Sl CLE3
10
8
6
4
2
0
A R YS MS OS YL ML FB FL F1 F2 F3 MG BF TF RF

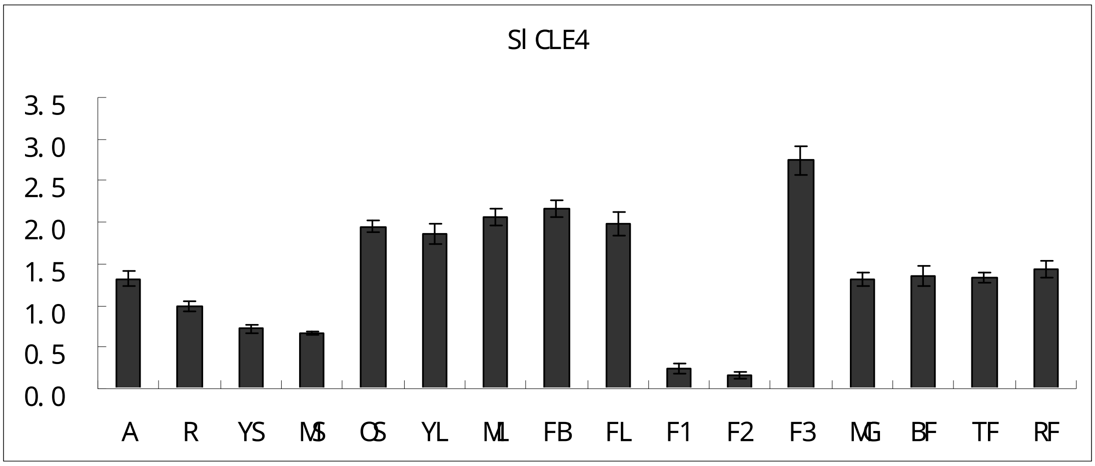
Sl CLE4
3.5
3.0
2.5
2.0
1.5
1.0
0.5
0.0
A R YS MS OS YL ML FB FL F1 F2 F3 MG BF TF RF

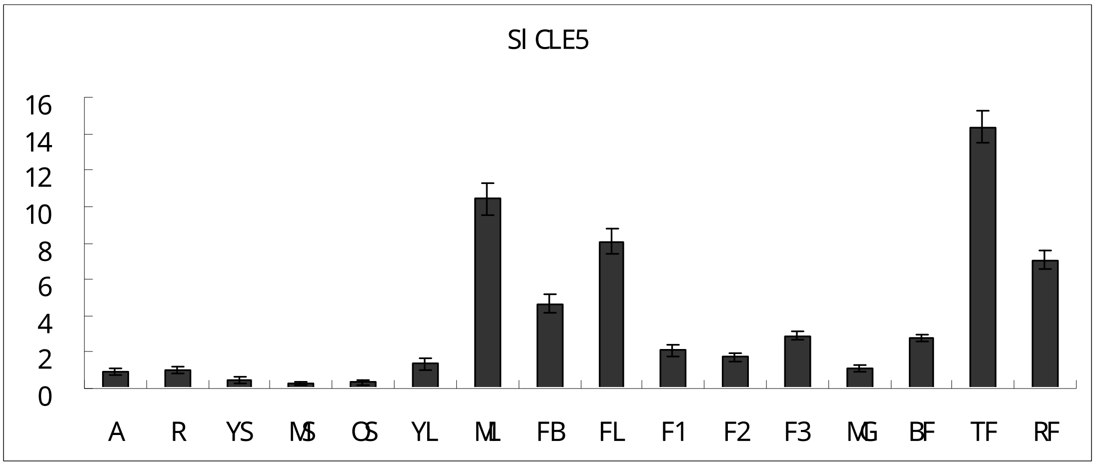
Sl CLE5
16
14
12
10
8
6
4
2
0
A R YS MS OS YL ML FB FL F1 F2 F3 MG BF TF RF

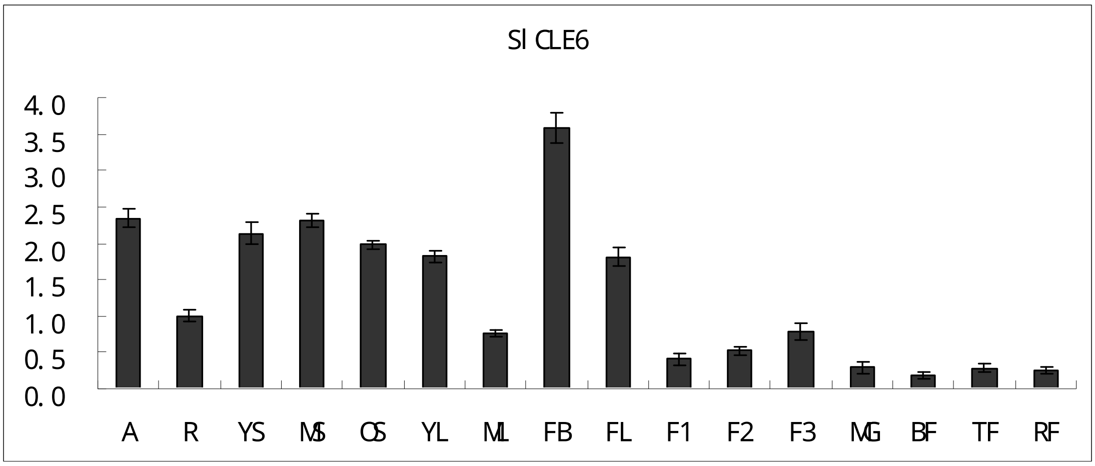
Sl CLE6
4.0
3.5
3.0
2.5
2.0
1.5
1.0
0.5
0.0
A R YS MS OS YL ML FB FL F1 F2 F3 MG BF TF RF

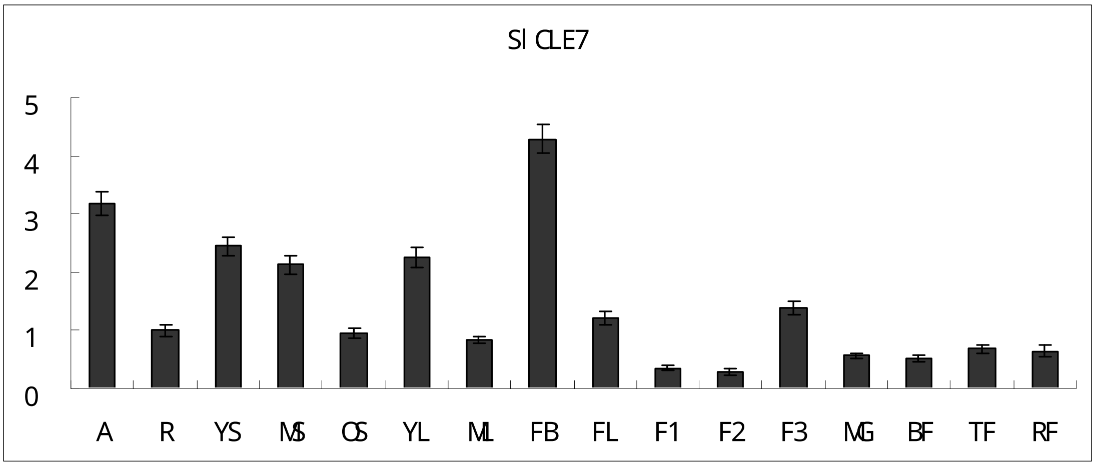
Sl CLE7
5
4
3
2
1
0
A
R
YS
MS
OS
YL
ML
FB
FL
F1
F2
F3
MG
BF
TF
RF

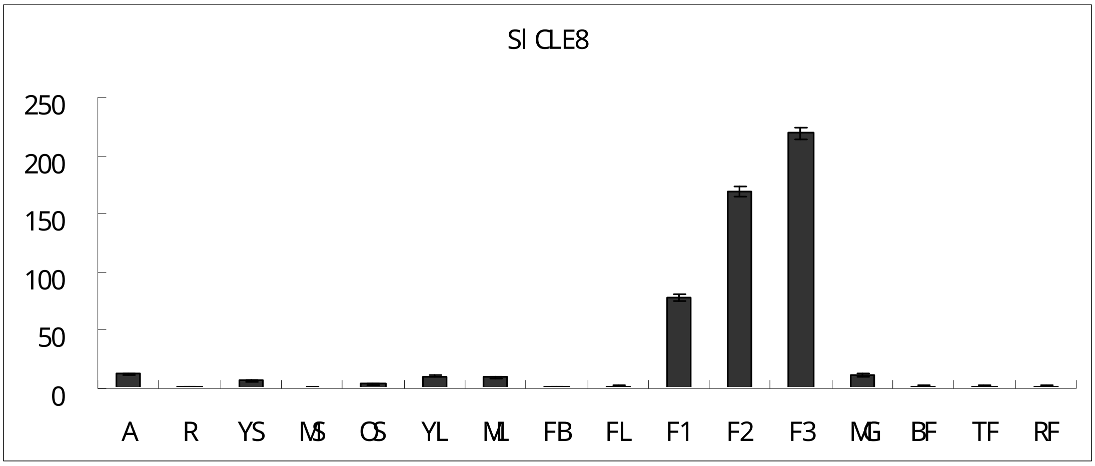
Sl CLE8
250
200
150
100
50
0
A
R
YS
MS
OS
YL
ML
FB
FL
F1
F2
F3
MG
BF
TF
RF

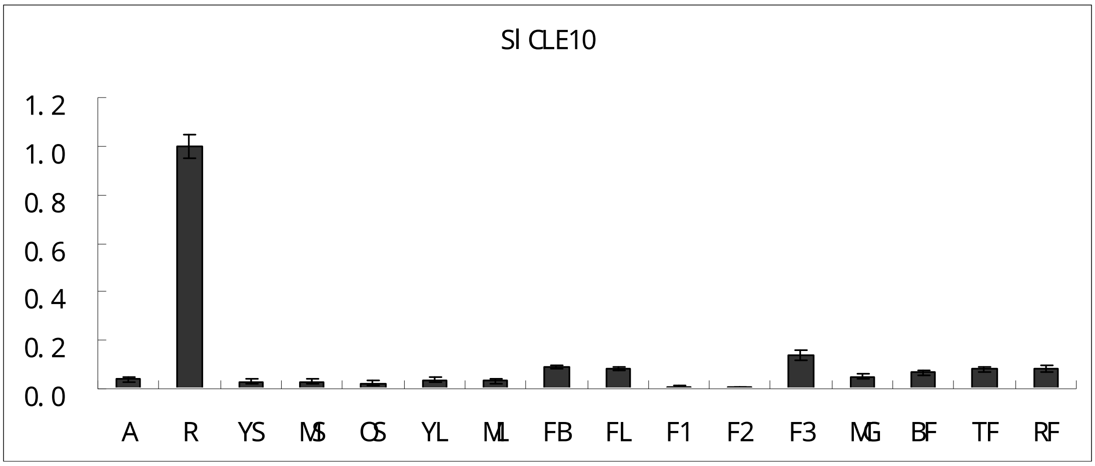
Sl CLE10
1.2
1.0
0.8
0.6
0.4
0.2
0.0
A
R
YS
MS
OS
YL
ML
FB
FL
F1
F2
F3
MG
BF
TF
RF

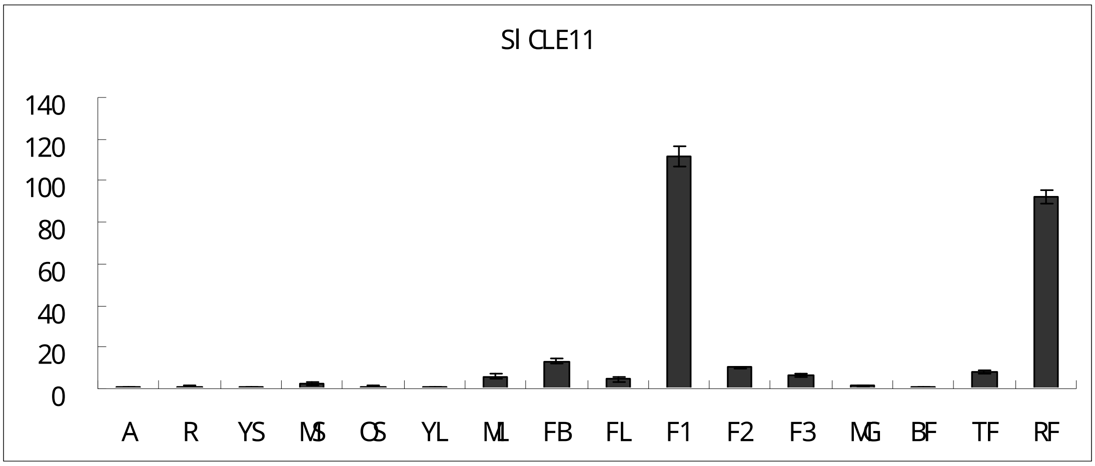

Sl CLE11
140
120
100
80
60
40
20
0
A R YS MS OS YL ML FB FL F1 F2 F3 MG BF TF RF

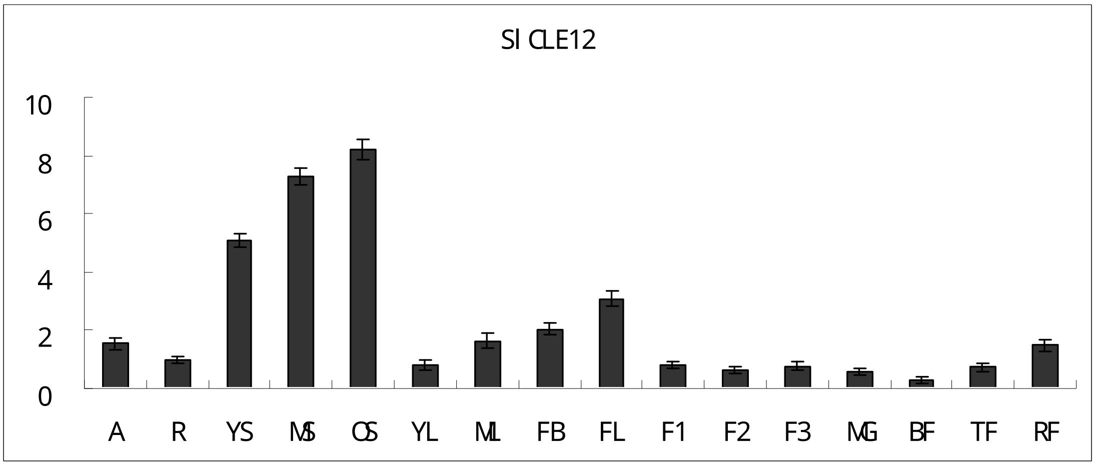

Sl CLE12
10
8
6
4
2
0
A R YS MS OS YL ML FB FL F1 F2 F3 MG BF TF RF

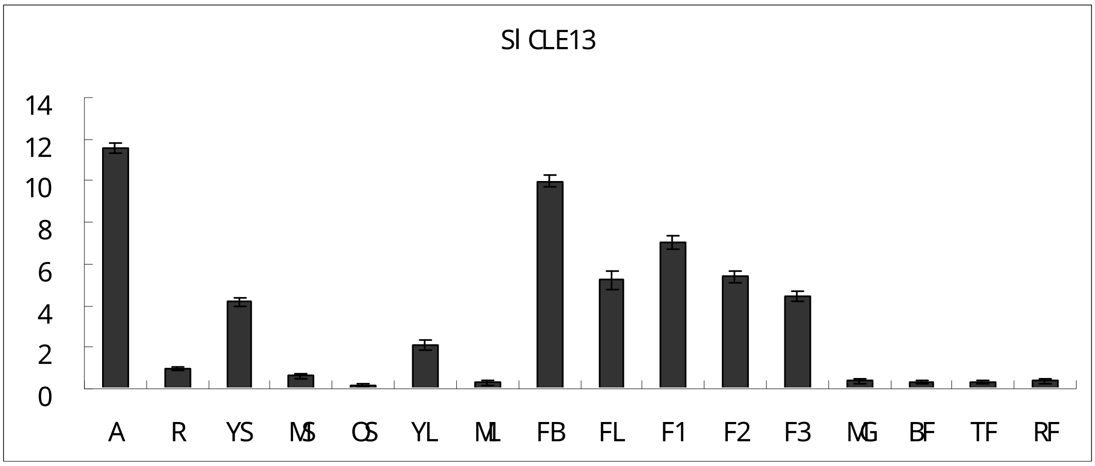

Sl CLE13
14
12
10
8
6
4
2
0
A R YS MS OS YL ML FB FL F1 F2 F3 MG BF TF RF

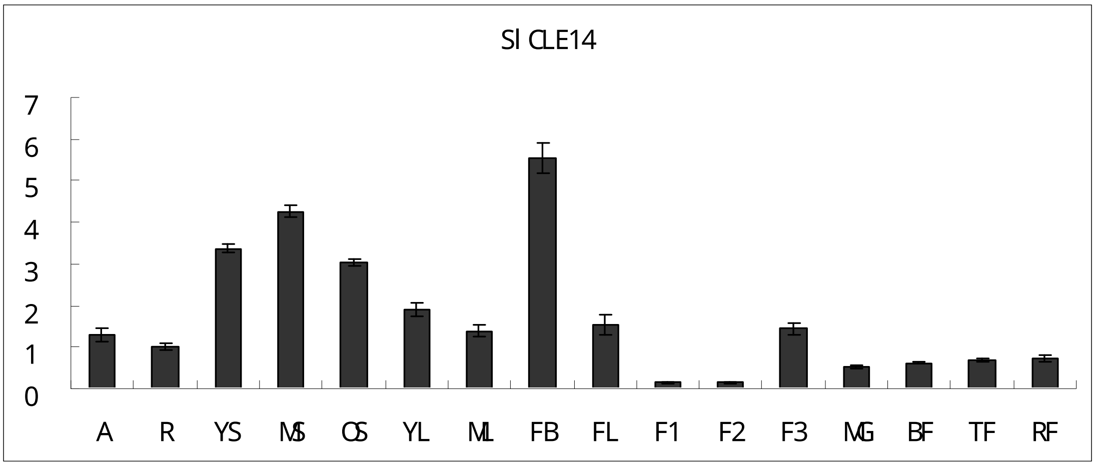
Sl CLE14
7
6
5
4
3
2
1
0
A
R
YS
MS
OS
YL
ML
FB
FL
F1
F2
F3
MG
BF
TF
RF

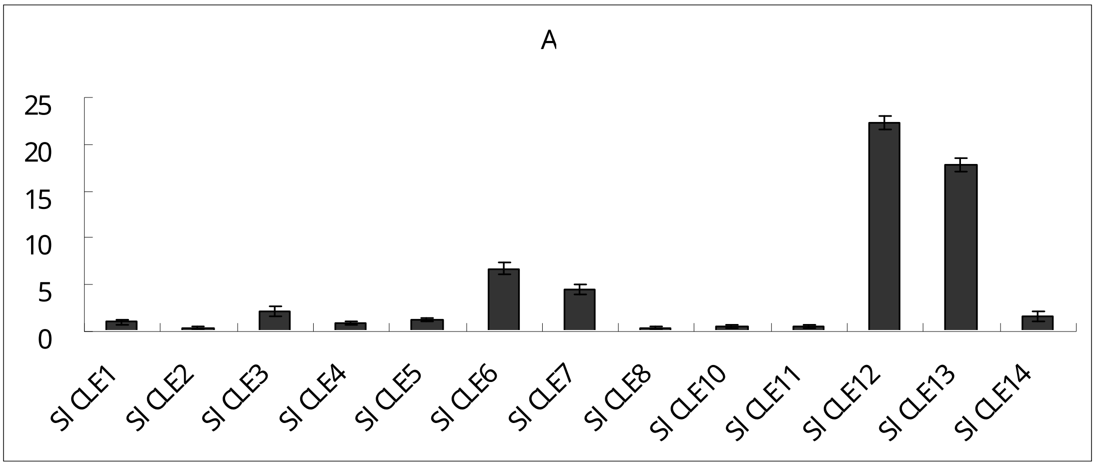
A
25
20
15
10
5
0
Sl CLE1
Sl CLE2
Sl CLE3
Sl CLE4
Sl CLE5
Sl CLE6
Sl CLE7
Sl CLE8
Sl CLE10
Sl CLE11
Sl CLE12
Sl CLE13
Sl CLE14

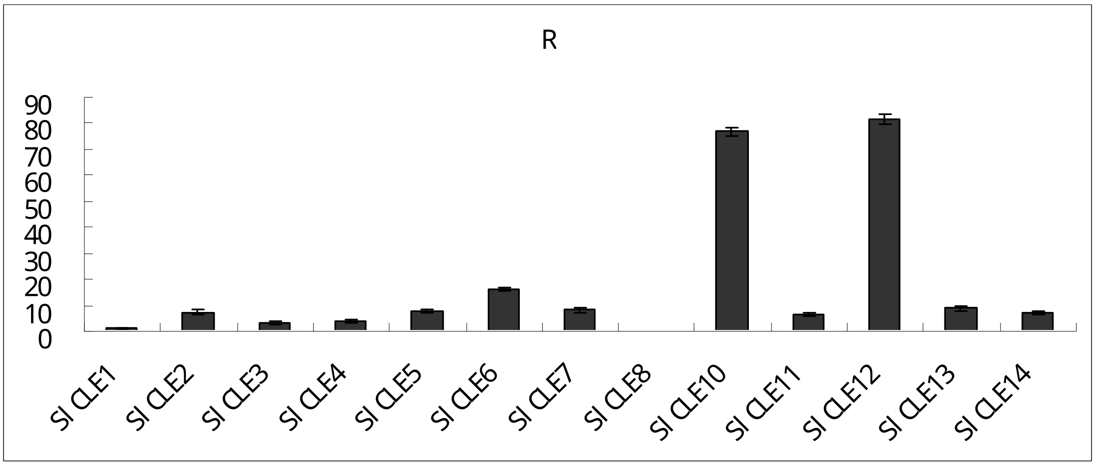
R
90
80
70
60
50
40
30
20
10
0
Sl CLE1
Sl CLE2
Sl CLE3
Sl CLE4
Sl CLE5
Sl CLE6
Sl CLE7
Sl CLE8
Sl CLE10
Sl CLE11
Sl CLE12
Sl CLE13
Sl CLE14

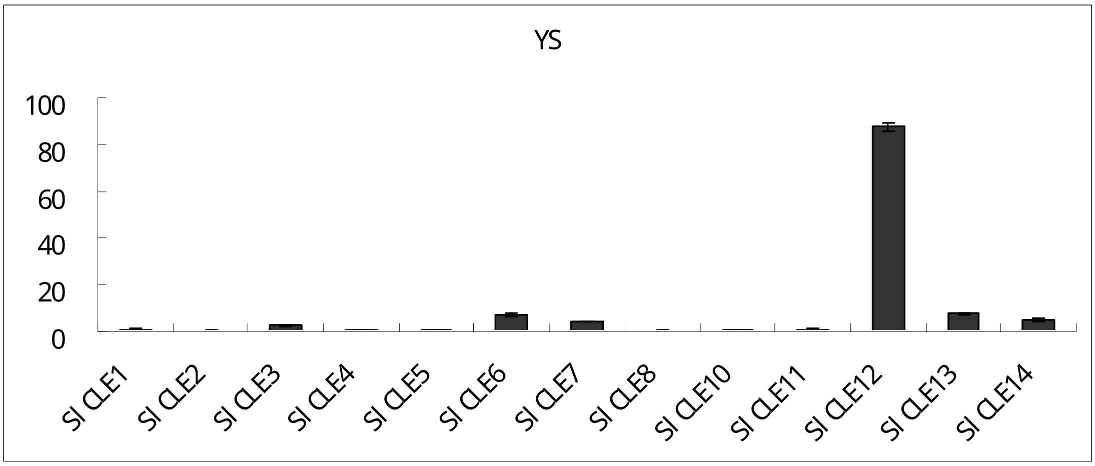
YS
100
80
60
40
20
0
Sl CLE1
Sl CLE2
Sl CLE3
Sl CLE4
Sl CLE5
Sl CLE6
Sl CLE7
Sl CLE8
Sl CLE10
Sl CLE11
Sl CLE12
Sl CLE13
Sl CLE14

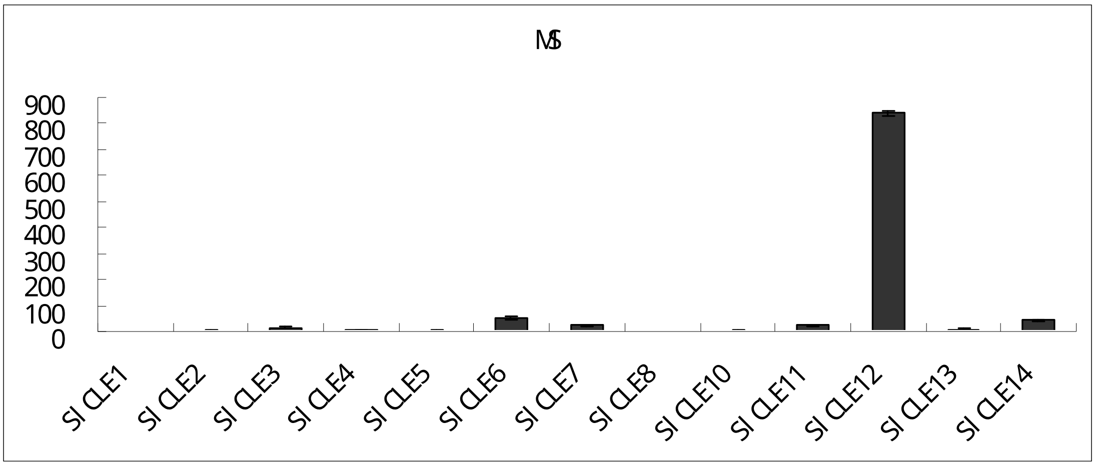

MS
900
800
700
600
500
400
300
200
100
0
Sl CLE1
Sl CLE2
Sl CLE3
Sl CLE4
Sl CLE5
Sl CLE6
Sl CLE7
Sl CLE8
Sl CLE10
Sl CLE11
Sl CLE12
Sl CLE13
Sl CLE14

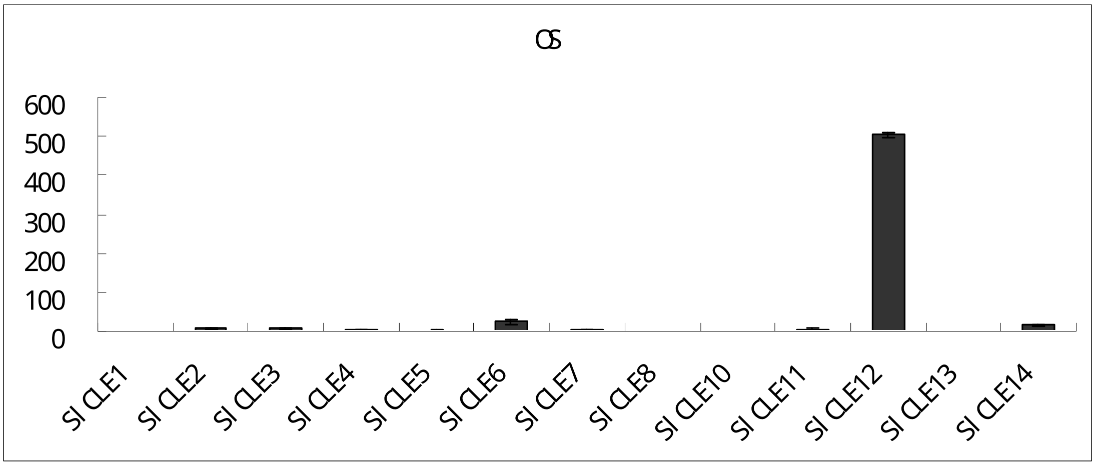

OS
600
500
400
300
200
100
0
Sl CLE1
Sl CLE2
Sl CLE3
Sl CLE4
Sl CLE5
Sl CLE6
Sl CLE7
Sl CLE8
Sl CLE10
Sl CLE11
Sl CLE12
Sl CLE13
Sl CLE14

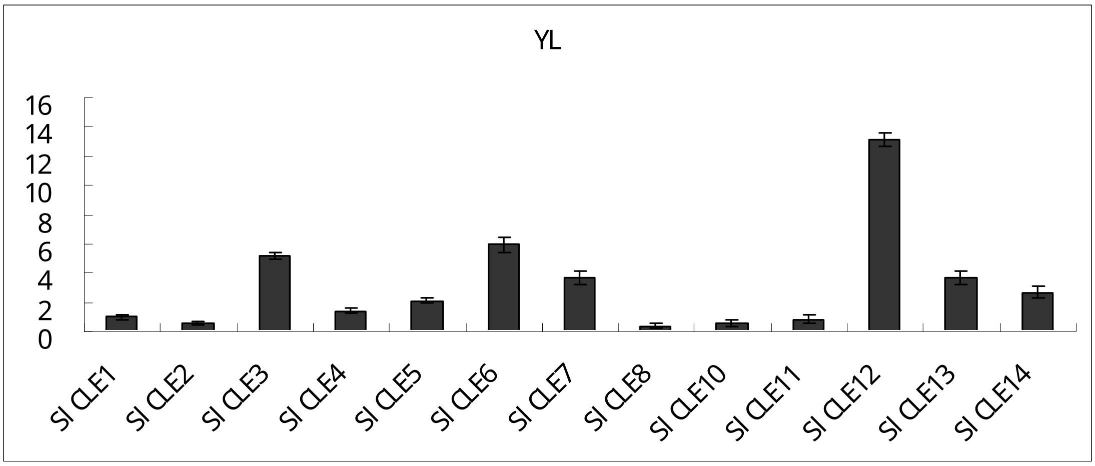

YL
16
14
12
10
8
6
4
2
0
Sl CLE1
Sl CLE2
Sl CLE3
Sl CLE4
Sl CLE5
Sl CLE6
Sl CLE7
Sl CLE8
Sl CLE10
Sl CLE11
Sl CLE12
Sl CLE13
Sl CLE14

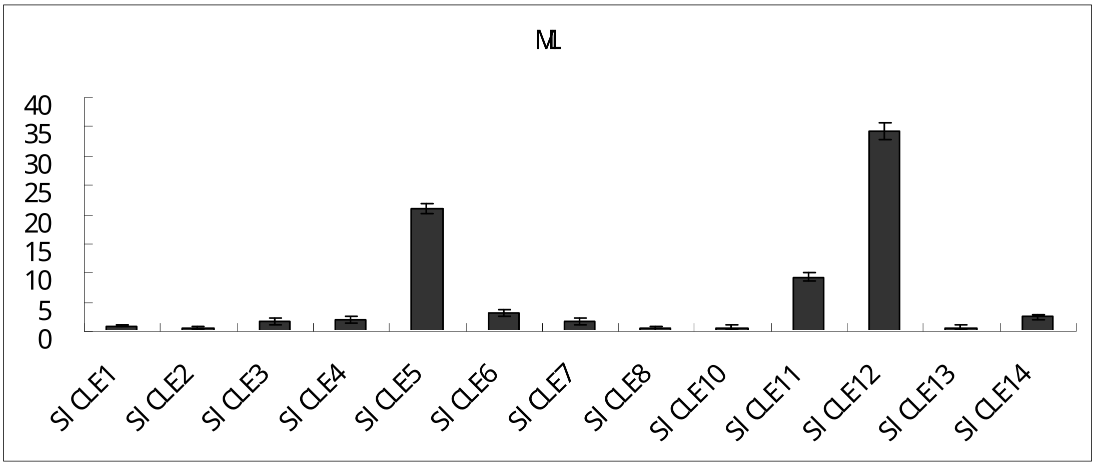
ML
40
35
30
25
20
15
10
5
0
Sl CLE1
Sl CLE2
Sl CLE3
Sl CLE4
Sl CLE5
Sl CLE6
Sl CLE7
Sl CLE8
Sl CLE10
Sl CLE11
Sl CLE12
Sl CLE13
Sl CLE14

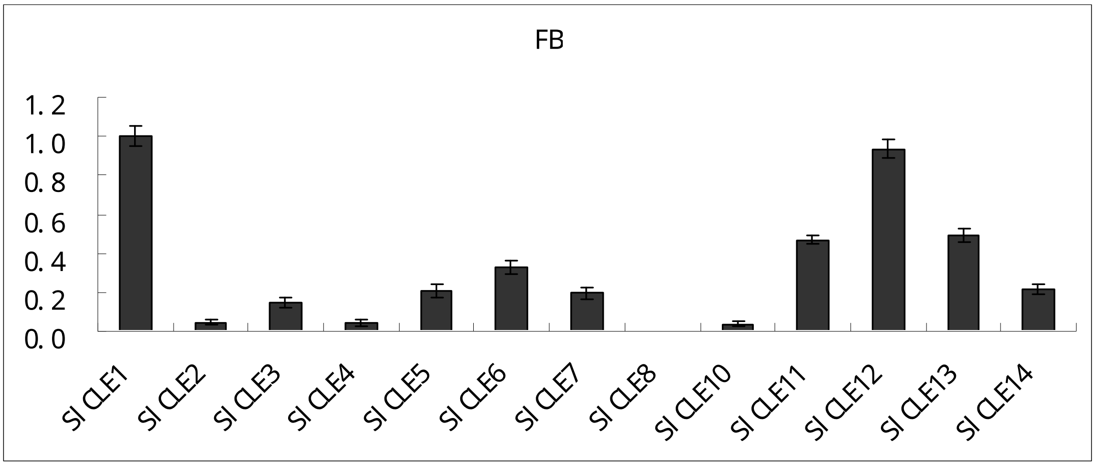
FB
1. 2
1. 0
0. 8
0. 6
0. 4
0. 2
0. 0
Sl CLE1
Sl CLE2
Sl CLE3
Sl CLE4
Sl CLE5
Sl CLE6
Sl CLE7
Sl CLE8
Sl CLE10
Sl CLE11
Sl CLE12
Sl CLE13
Sl CLE14

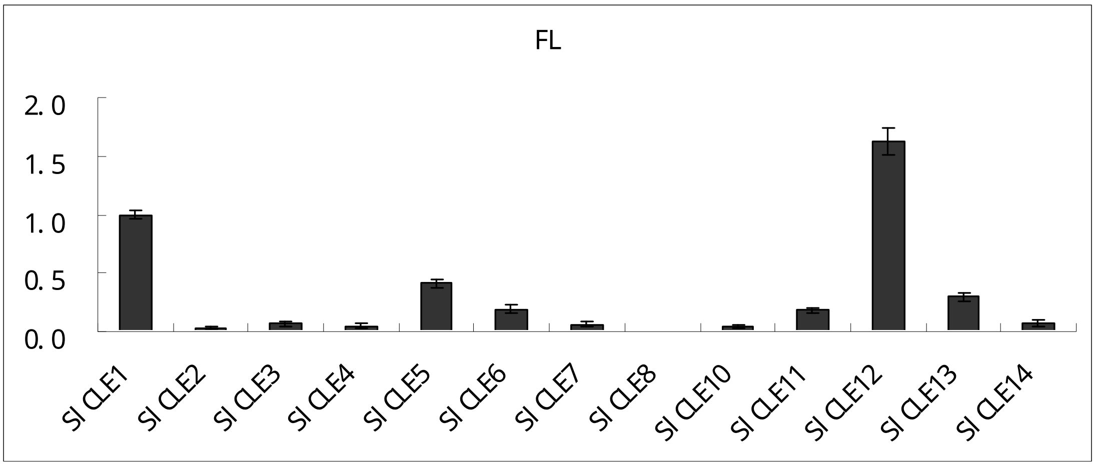
FL
2. 0
1. 5
1. 0
0. 5
0. 0
Sl CLE1
Sl CLE2
Sl CLE3
Sl CLE4
Sl CLE5
Sl CLE6
Sl CLE7
Sl CLE8
Sl CLE10
Sl CLE11
Sl CLE12
Sl CLE13
Sl CLE14

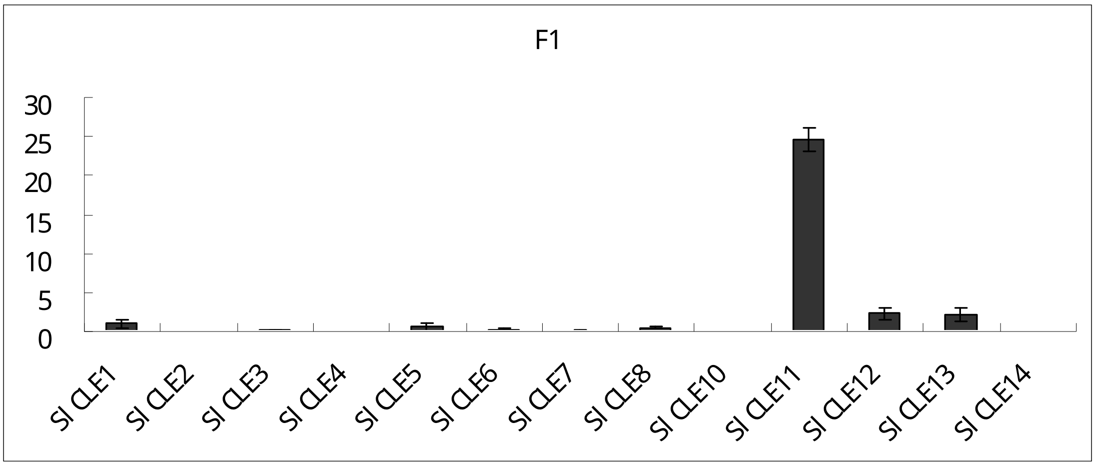
F1
30
25
20
15
10
5
0
Sl CLE1
Sl CLE2
Sl CLE3
Sl CLE4
Sl CLE5
Sl CLE6
Sl CLE7
Sl CLE8
Sl CLE10
Sl CLE11
Sl CLE12
Sl CLE13
Sl CLE14

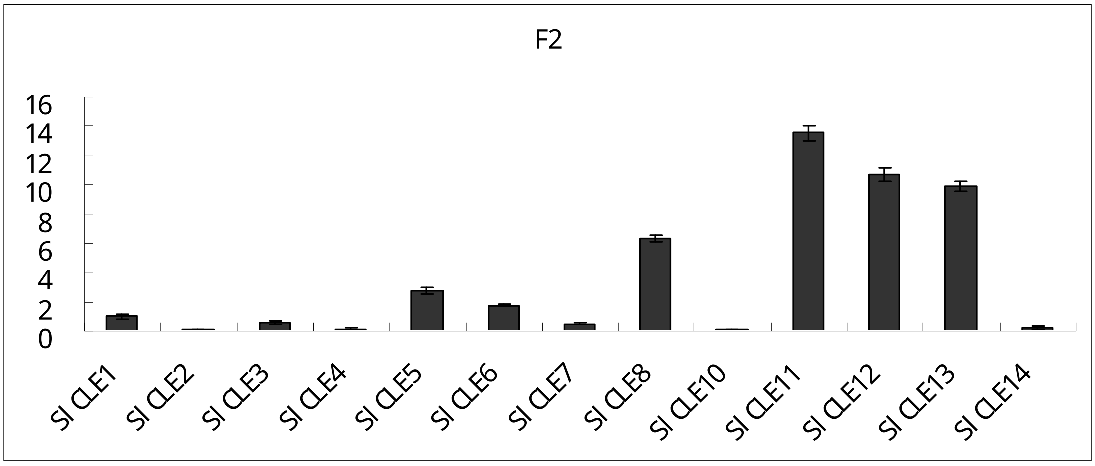
F2
16
14
12
10
8
6
4
2
0
Sl CLE1
Sl CLE2
Sl CLE3
Sl CLE4
Sl CLE5
Sl CLE6
Sl CLE7
Sl CLE8
Sl CLE10
Sl CLE11
Sl CLE12
Sl CLE13
Sl CLE14

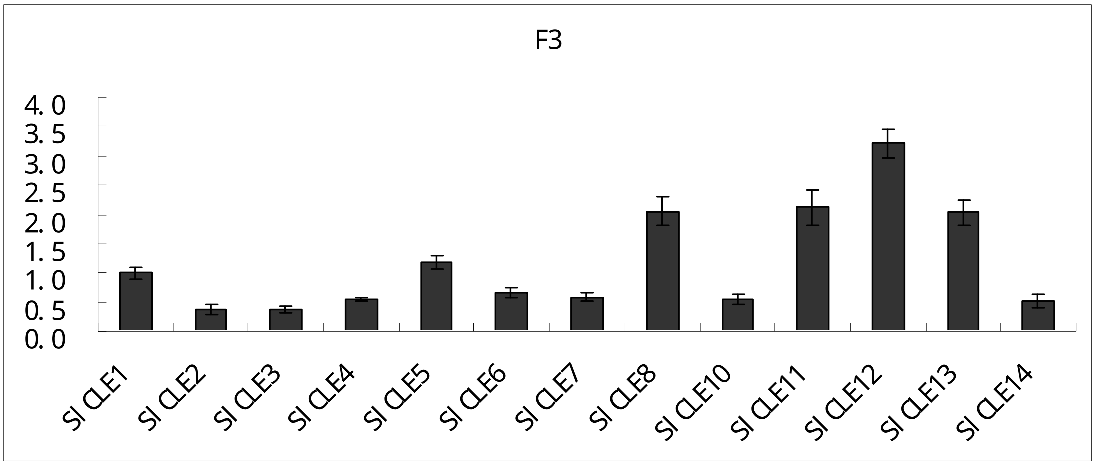
F3
4.0
3.5
3.0
2.5
2.0
1.5
1.0
0.5
0.0
Sl CLE1
Sl CLE2
Sl CLE3
Sl CLE4
Sl CLE5
Sl CLE6
Sl CLE7
Sl CLE8
Sl CLE10
Sl CLE11
Sl CLE12
Sl CLE13
Sl CLE14

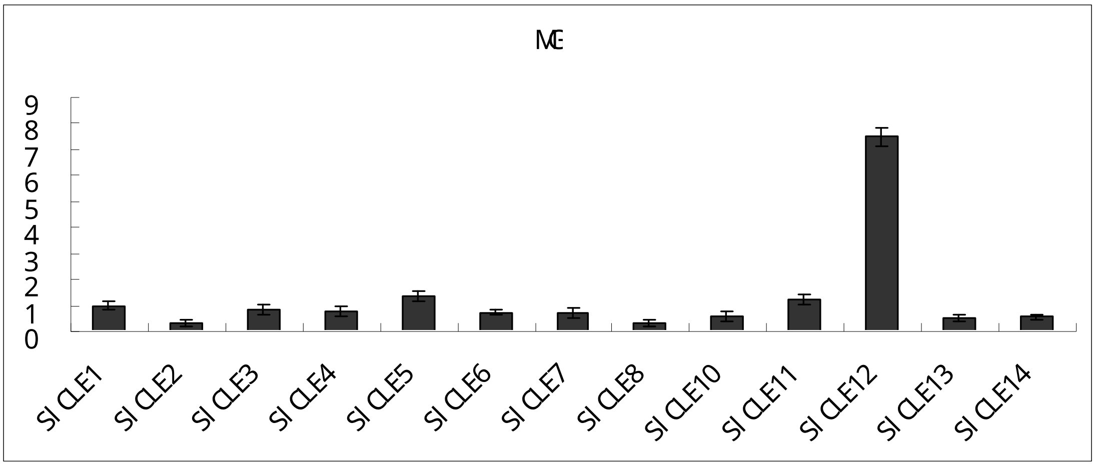

MG
9
8
7
6
5
4
3
2
1
0
Sl CLE1
Sl CLE2
Sl CLE3
Sl CLE4
Sl CLE5
Sl CLE6
Sl CLE7
Sl CLE8
Sl CLE10
Sl CLE11
Sl CLE12
Sl CLE13
Sl CLE14

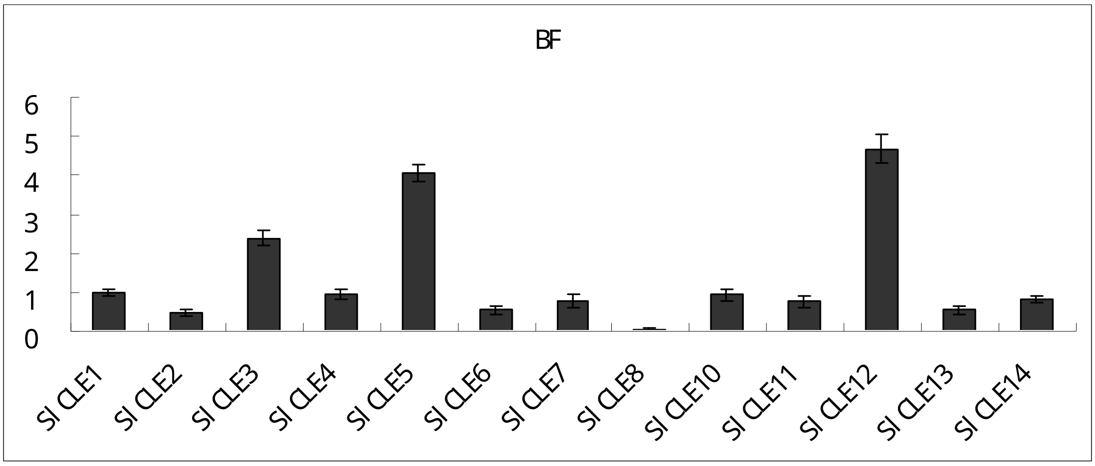

BF
6
5
4
3
2
1
0
Sl CLE1
Sl CLE2
Sl CLE3
Sl CLE4
Sl CLE5
Sl CLE6
Sl CLE7
Sl CLE8
Sl CLE10
Sl CLE11
Sl CLE12
Sl CLE13
Sl CLE14

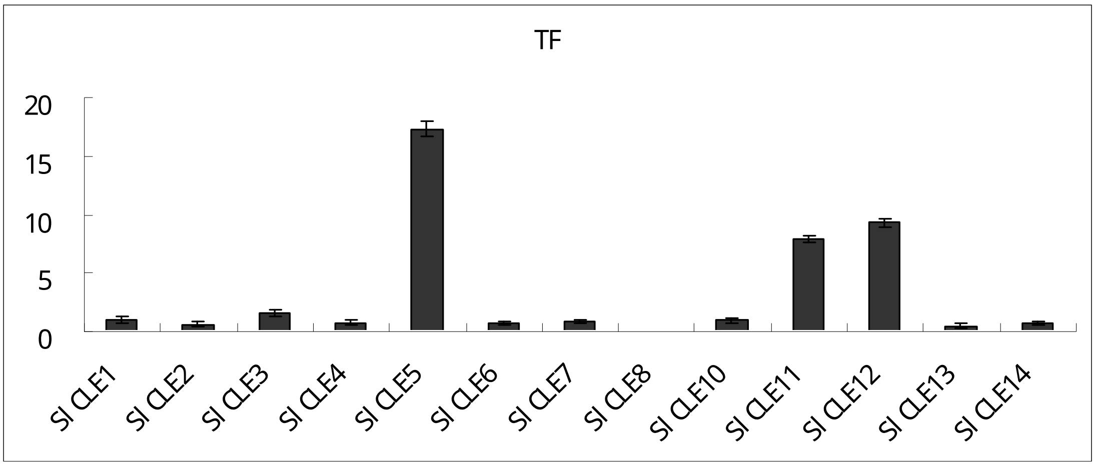

TF
20
15
10
5
0
Sl CLE1
Sl CLE2
Sl CLE3
Sl CLE4
Sl CLE5
Sl CLE6
Sl CLE7
Sl CLE8
Sl CLE10
Sl CLE11
Sl CLE12
Sl CLE13
Sl CLE14

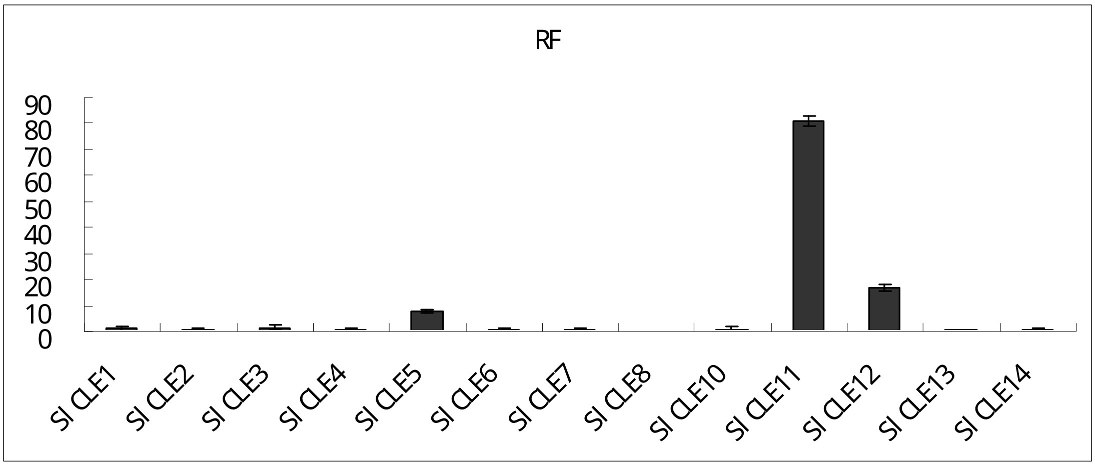

RF
90
80
70
60
50
40
30
20
10
0
Sl CLE1
Sl CLE2
Sl CLE3
Sl CLE4
Sl CLE5
Sl CLE6
Sl CLE7
Sl CLE8
Sl CLE10
Sl CLE11
Sl CLE12
Sl CLE13
Sl CLE14
